# Analysis of potential roles of combinatorial microRNA regulation in occurrence of valvular heart disease with atrial fibrillation based on computational evidences

**DOI:** 10.1371/journal.pone.0221900

**Published:** 2019-09-03

**Authors:** Guangbin Wang, Nini Rao, Dingyun Liu, Hongxiu Jiang, Ke Liu, Feng Yang, Yangwei Chen, Keli Huang

**Affiliations:** 1 Center for Informational Biology, School of Life Science and Technology, University of Electronic Science and Technology of China, Chengdu, China; 2 School of Life Science and Technology, University of Electronic Science and Technology of China, Chengdu, China; 3 Key Laboratory for NeuroInformation of the Ministry of Education, University of Electronic Science and Technology of China, Chengdu, China; 4 Institute of Electronic and Information Engineering of UESTC in Guangdong, Dongguan, China; 5 Subsidiary Hospital, University of Electronic Science and Technology of China, Chengdu, China; The University of British Columbia, NEW ZEALAND

## Abstract

**Background:**

Atrial fibrillation (AF) is the most common arrhythmia. Patients with valvular heart disease (VHD) frequently have AF. Growing evidence demonstrates that a specifically altered pattern of microRNA (miRNA) expression is related to valvular heart disease with atrial fibrillation (AF-VHD) processes. However, the combinatorial regulation by multiple miRNAs in inducing AF-VHD remains largely unknown.

**Methods:**

The work identified AF-VHD-specific miRNAs and their combinations through mapping miRNA expression profile into differential co-expression network. The expressions of some dysregulated miRNAs were measured by quantitative reverse transcription polymerase chain reaction (qRT-PCR). The regulations of signaling pathways by the combinatorial miRNAs were predicted by enrichment analysis tools.

**Results:**

Thirty-two differentially expressed (DE) miRNAs were identified to be AF-VHD-specific, some of which were new findings. These miRNAs interacted to form 5 combinations. qRT-PCR confirmed the different expression of several identified miRNAs, which illustrated the reliability and biomarker potentials of 32 dysregulation miRNAs. The biological characteristics of combinatorial miRNAs related to AF-VHD were highlighted. Twelve signaling pathways regulated by combinatorial miRNAs were predicted to be possibly associated with AF-VHD.

**Conclusions:**

The AF-VHD-related signaling pathways regulated by combinatorial miRNAs may play an important role in the occurrence of AF-VHD. The work brings new insights into biomarkers and miRNA combination regulation mechanism in AF-VHD as well as further biological experiments.

## Introduction

Atrial fibrillation (AF) is a highly prevalent disease with a significant genetic component, considering the most common sustained arrhythmia. Patients with valvular heart disease frequently have AF due to elevated pressure and dilatation of the left and right atria and pulmonary veins [1]. Compared to people with normal sinus rhythm (SR), patients who have AF and VHD (VHD with AF, or AF-VHD) are at a higher risk (17.5-fold) for stroke and have a four-fold higher incidence of embolism [2–3]. AF, resulting from the dysfunction of numerous molecular processes in the body that affects individual tissue responses to stimuli [4], contributes to worsening pathology of VHD. Thus, it is important to understand AF-VHD biology for diagnosis and treatment of the complex disease.

MicroRNAs (miRNAs) are small non-coding regulatory molecules of ~22nt length that modify gene expression at the post-transcriptional level by binding to the 3' untranslated region (UTRs) of their target messenger RNAs (mRNAs). As the central players in the regulation of gene expression, miRNAs participate in physiological processes, including cell differentiation, proliferation, mobility, metabolism, apoptosis and stress responses [5]. A single miRNA can regulate multiple mRNAs, and each mRNA may be a target of multiple miRNAs. Hence, the possible pathways for miRNA-dependent regulation are complicated [6–7]. In this model, a biological response occurs only after co-expression of various miRNAs that target different components of a functional network [8–9] or are required to repress a single target [10–11].

A specifically altered pattern of miRNA expression (the miRNA signature) has been confirmed to be associated with AF-VHD processes [12–14]. Complex diseases are not caused by miRNAs which act alone but by highly interacting miRNA networks (combinations), which result from genetic and environmental perturbations ultimately driving phyiological states toward disease [15–18]. This suggests that the progression of AF-VHD are associated with the combinational work of multiple miRNAs. The differential co-expression network can be used to identify the changes between disease and the control through mapping molecular expression profiles into co-expression networks, can reveal context-specific interactions and filter out unspecific dominant interaction among molecules [19]. Therefore, the work first identified AF-VHD-specific miRNAs and then found the combinations among them through constructing miRNA differential co-expression network using computational methods. Next, the target genes of each combinatorial microRNAs were predicted and the signaling pathways regulated by each combination of microRNAs were enriched on Kyoto Encyclopedia of Genes and Genomes (KEGG) database using its target genes. The potential roles of these signaling pathways in the occurrence of AF-VHD were finally analyzed based on the computational evidences.

## Materials and methods

### Data

The miRNA expression data was downloaded from GEO (http://www.ncbi.nlm.nih.gov/geo/) (GEO accession no. GSE28954), where there are 8 AF-VHD patients (as disease group) and 9 VHD patients with Sinus rhythm (as control group). This data was used to screen specific miRNAs associated with AF-VHD and construct differential co-expression networks. Wherein, all the samples consist of tissues from right and left atrial (RA and LA) myocardium (appendage). There is no identification of 3p or 5p in this miRNA expression data.

One hundred and sixty-nine differential expression (DE) genes associated with AF-VHD were from the research results of Lamiault et al. [20], and used to decrease the false positives of DE miRNAs between AF-VHD and VHD sample groups.

### Tissue samples

We collected the atrial appendage tissues of 6 AF-VHD patients and 6 VHD patients for qRT-PCR experiments to validate the differential expression of partial DE miRNAs. The patients satisfied the following criteria for inclusion and exclusion:

Inclusion: The patient's age ranges from 40 to 70 years, regardless of gender. The detailed electrocardiograph and echocardiography recordings verified that VHD patients had rheumatic VHD, in which mitral valve disease was recommended as the main disease. AF-VHD patients conformed to the diagnostic criteria for valvular heart disease combined with atrial fibrillation, in which AF with mitral valve disease was recommended as the main disease, and the AF had lasted for more than one year.

Exclusion: (1) Patients whose expected survival time was less than 1 year because of other serious diseases. (2) Patients who disagree with the follow-up. (3) AF was due to a reversible cause, including acute myocardial infarction, acute myocarditis and hyperthyroidism without treatment. AF was caused by electrophysiological examination, angiography and pacemaker implantation or cardiothoracic surgery recently, but it did not reappear after treatment. (4) Persistent AF did not relapse after medication or electrical cardioversion. (5) Patients after prosthetic valve replacement treatment. (6) Patients with lone AF. (7) Patients with systolic or diastolic blood pressure ≥ 180/100 mm Hg. But the patients could be included when their blood pressure was under control. (8) Infected patients. (9) Patients with severe liver or kidney diseases, or chronic renal failure with serum creatinine > 186 mg/dl. Patients whose transaminase exceeded the reference value by more than 1.5 times.

This experiment was approved by the Ethics review board of school of Life Science and Technology in University of Electronic Science and Technology of China (UESTC), Chengdu, China, and all patients gave informed consents.

Atrial appendage tissue was collected from VHD and AF-VHD patients undergoing heart surgery at the Affiliated Hospital, Medicine School, UESCT, Chengdu, China. In all cases, tissues removed at surgery were placed immediately in ice-cold saline. Surgically removed tissues (the thickness of less than 0.5cm) were immersed in the tubes containing RNA later solution (Invitrogen) within 10 min of excision and then they were stored at -4°C all night. RNAlater was removed after overnight treatment and the samples were stored at -80°C.

### Identifying DE miRNAs bewteen AF-VHD and VHD samples

This work includes three steps. Because the sample number of miRNA expression data is small, the asymmetric principal component analysis (APCA)-Bootstrap algorithm [14] was firstly selected to identify DE miRNAs in AF-VHD tissues compared with VHD tissues from microRNA expression data. Secondly, TargetScan [21], miRanda [22] and PicTar [23] were used to predict the target genes of each DE miRNA. Only those target genes in all three databases were retained. Thirdly, the genes associated with AF-VHD [20] were used to exclude the false positives of DE miRNAs identified in the first step. Only those DE miRNAs targeting the AF-VHD-related genes were retained.

### Constructing differential co-expression networks

Using the identified DE miRNAs, we constructed the two co-expression networks corresponding to AF-VHD and VHD, respectively, according to Pearson correlation coefficients (PCCs) between the expression values of pairwise DE miRNAs across the samples in a sample group. Wherein, a DE miRNA pair is connected only when PCC>0.9 [24] between their expression values. Therefore, a edge between two nodes (two DE miRNAs) in two co-expression networks indicates that two connected DE miRNAs have co-expression relationship.

Although the two co-expression networks have same nodes (DE miRNAs), their connection edges are different because there are expression difference between two sample groups. The work introduced the change of connection degree (*K*) of a DE miRNA to reveal this difference between the co-expression networks of AF-VHD and VHD. Therefore, the *K* can be expressed by the number of differential connecting edges of a DE miRNA (node) in AF-VHD co-expression networks compared with that of corresponding miRNA in VHD co-expression network.

Supposing that the number of identified DE miRNAs is *M*, and the change of connection degree of the *i-*th DE miRNA is *K*(*i*) (*i* = 1,2,…,*M)*, *K*(*i*) can be calculated as follows:

$$k\left(i\right)=\sum_{j}R_{\mathrm{AF}-\mathrm{VHD}}\left(i,j\right)⊕R_{\mathrm{VHD}}\left(i,j\right),i,j=1,2,\cdots ,M,j\neq i$$ (1)

where *R*_AF-VHD_**(***i*, *j***)** is the quantified PCC between the *i-*th DE miRNA and the *j-*th DE miRNA (*j* = 1,2,···, *M*) in AF-VHD co-expression network. The value of *R*_AF-VHD_ (*i*, *j*) can be quantified as “1” when PCC≧0.9, and as “0” when PCC<0.9 because the threshold connecting a edge between two DE miRNAs is 0.9 [24]. Similarly, *R*_VHD_**(***i*, *j***)** is the quantified PCC between the same pair of DE miRNAs in VHD co-expression network. If *K*(*i*)≠0 of the *i-*th DE miRNA, the connection degree of the *i-*th DE miRNA has the difference between AF-VHD and VHD co-expression networks. Inversely, if *K*(*i*) = 0 of the *i-*th DE miRNA, the co-expression relationship of the *i-*th DE miRNA has not changed in AF-VHD and VHD co-expression networks. When we cancel those DE miRNA nodes with *K* = 0 from AF-VHD co-expression network, the left networks, consisting of those DE miRNA nodes with *K* ≠ 0 and the correponding edges, reveals the changes that the co-expression network of AF-VHD differs from that of VHD. Therefore, it is named the differential co-expression networks.

### Identifying AF-VHD-specific miRNAs and their combinations

In the differential co-expression network, the nodes (DE miRNAs) show the expression difference between AF-VHD and VHD sample groups, and the connection degree of a node (DE miRNA) indicates the co-expression difference within AF-VHD and VHD sample groups. Therefore, these miRNAs in the differential co-expression network are identified as the AF-VHD-specific miRNAs. The combinatorial relationships of these dysregulated miRNAs are determined by the connection relationships in the differential co-expression network.

To confirm the findings identified by our method, several dysregulated miRNAs were randomly selected to measure their expressions using qRT-PCR. The RT reaction used MMLV reverse transcriptase (Epicentre, Madison, WI). The qPCR was performed by a ViiA 7 Real-time PCR System (Applied Biosystems, Foster City, CA). The qPCR reaction cycles were as follows: 1 cycle of 10 min at 95°C; 40 cycles of 10 s at 95°C, and of 60 s at 60°C. The relative expression level of each miRNA was normalized to the internal control of small nuclear U6 expression, calculated by the 2 ^- **ΔΔ**CT^ method.

### Enriching biological features of combinatorial miRNAs and signaling pathways regulated

We first predicted the target genes regulated by the combinations of miRNAs. The target genes of each miRNA in a combination were predicted by TargetScan, miRanda and PicTar databases, respectively. Only those genes that appeared in three databases at the same time were retained as the target genes of one miRNA in a combination. Those genes, commonly regulated by all the miRNAs in a combination, were retained as the target genes regulated by this combination. Using the predicted target genes of a combinatorial miRNAs, the biological features of this combination were enriched by Gene Ontology (GO) in DAVID (http://david.abcc.ncifcrf.gov/).

Moreover, the regulations of signaling pathways by a combination of miRNAs were enriched by Kyoto Encyclopedia of Genes and Genomes (KEGG) in DAVID.

## Results

### Identified AF-VHD-specific miRNAs

We identified 47 DE miRNAs from the miRNA expression data (See S1 Table). S2 Table shows the normalized PCCs between pairwise DE miRNAs corresponding to AF-VHD and VHD samples, reflecting the connection relationships among 47 DE miRNAs in two co-expression networks. Eq (1) was used to calculate the change of connection degree of each DE miRNA between AF-VHD and VHD (See S1 Table).

The connection degrees of the 32 miRNAs have been changed, so these 32 miRNAs are identified as AF-VHD-specific miRNAs (See Table 1). Among them, hsa-miR-1, hsa-miR-101, hsa-miR-133a, hsa-miR-133b, hsa-miR-21, hsa-miR-29b, hsa-miR-30a, hsa-miR-30d and hsa-miR-30e (marked by bold in Table 1) have been illustrated to be associated with AF-VHD [14]. Thus, some of our identification results were new findings.

**Table 1:** 32 AF-VHD-specific miRNAs and the changes of connection degrees.

No.	miRNA symbol	The change of connection degree
1	hsa-let-7c	3
2	hsa-let-7f	2
**3**	**hsa-miR-1**	**3**
**4**	**hsa-miR-101**	**4**
5	hsa-miR-10b	3
6	hsa-miR-1238	2
7	hsa-miR-1260	1
**8**	**hsa-miR-133a**	**7**
**9**	**hsa-miR-133b**	**3**
10	hsa-miR-136	2
11	hsa-miR-15b	1
12	hsa-miR-16	1
13	hsa-miR-181b	1
14	hsa-miR-208a	2
**15**	**hsa-miR-21**	**1**
16	hsa-miR-214	1
17	hsa-miR-22	5
18	hsa-miR-27b	3
**19**	**hsa-miR-29b**	**4**
**20**	**hsa-miR-30a**	**2**
**21**	**hsa-miR-30d**	**3**
**22**	**hsa-miR-30e**	**5**
23	hsa-miR-32	1
24	hsa-miR-335	1
25	hsa-miR-361-3p	2
26	hsa-miR-362-5p	1
27	hsa-miR-365	2
28	hsa-miR-370	2
29	hsa-miR-378	2
30	hsa-miR-409-3p	1
31	hsa-miR-410	2
32	hsa-miR-98	1

miRNAs marked by bold have been confirmed to be associated with AF-VHD.

### Differential co-expression network and combinations of miRNAs

**Fig 1:**
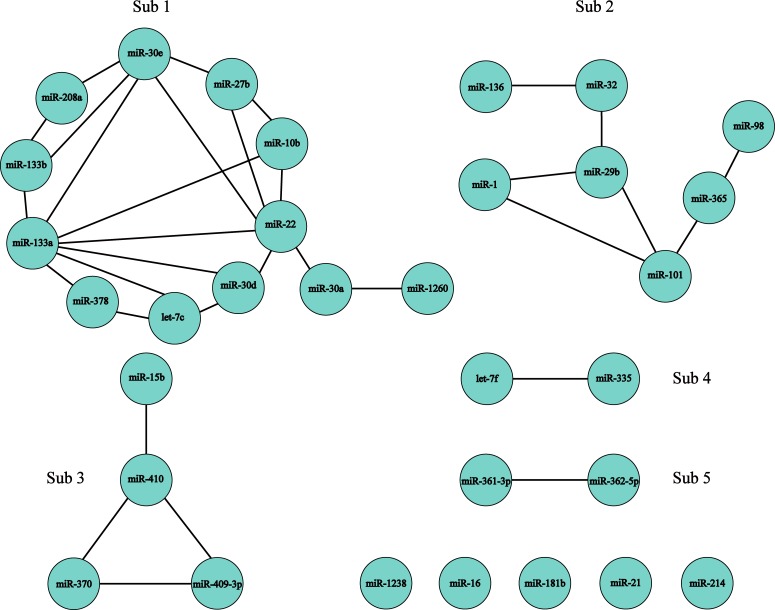
Differential co-expression network with 32 AF-VHD-specific miRNAs as the nodes.

When canceling those miRNAs with *K* = 0 from 47 DE miRNAs, we constructed the differential co-expression network (See Fig 1). The nodes of this network are 32 AF-VHD-specific miRNAs (See Table 1).

The network of Fig 1 is made up of 5 sub-networks and 5 isolated miRNAs—hsa-miR-1238, hsa-miR-16, hsa-miR-181b, hsa-miR-21 and hsa-miR-214, while each sub-network consists of interacting miRNAs and so is defined as a combination of miRNAs. For the sake of simplicity, the Sub 1 is used to represent the combination (the sub-network) formed by the interaction among 12 miRNAs—hsa-let-7c, hsa-miR-10b, hsa-miR-1260, hsa-miR-133a, hsa-miR-133b, hsa-miR-208a, hsa-miR-22, hsa-miR-27b, hsa-miR-30a, hsa-miR-30d, hsa-miR-30e and hsa-miR-410. The Sub 2 indicates the combination by interaction among 7 miRNAs—hsa-miR-1, hsa-miR-101, hsa-miR-136, hsa-miR-29b, hsa-miR-32, hsa-miR-365 and hsa-miR-98. The Sub 3 indicates the combination by interaction among hsa-miR-15b, hsa-miR-370, hsa-miR-409-3p and hsa-miR-410. The Sub 4 represents the combination by interaction between hsa-let-7f and hsa-miR-335. The Sub 5 indicates the combination by interactions between hsa-miR-361-3p and hsa-miR-362-5p.

### Validation of identified miRNAs

We randomly selected hsa-miR-30e, hsa-miR-32 and hsa-miR-98 from the two biggest combinatorial networks (Sub 1 and Sub 2) to validate the differential expression between the tissue samples of 6 AF-VHD and 6 VHD patients by qRT-PCR technology. Primers were chosen according to the name of a miRNA and the checked information on Microbase. Usually, the one with higher expression level in 3P and 5p was detected. S3 Table shows the RT primers and the primer sets specific for amplification of each miRNAs. The qRT-PCR experimental results (See S4 Table) showed that the average 2 ^- **ΔΔ**^ CT values of hsa-miR-30e, hsa-miR-32 and hsa-miR-98 in AF-VHD group were 0.568 (P<0.0047), 0.494 (P<0.0022) and 0.485 (P<0.0016) times those in VHD control group, respectively. Thus three critical miRNAs were down regulated in AF-VHD groups. It shows the different expression and reliability of identified miRNAs.

### Biological features of combinatorial miRNAs related to AF-VHD

The Sub 1, Sub 2, Sub 3 and Sub 4 were predicted to regulate 881, 528, 47, and 62 target genes (S5 Table), respectively, but Sub 5 has no predictable common target genes in TargetScan, miRanda and PicTar databases. Therefore, the biological features only for Sub1, Sub 2, Sub 3 and Sub 4 were subsequently analyzed.

**Fig 2:**
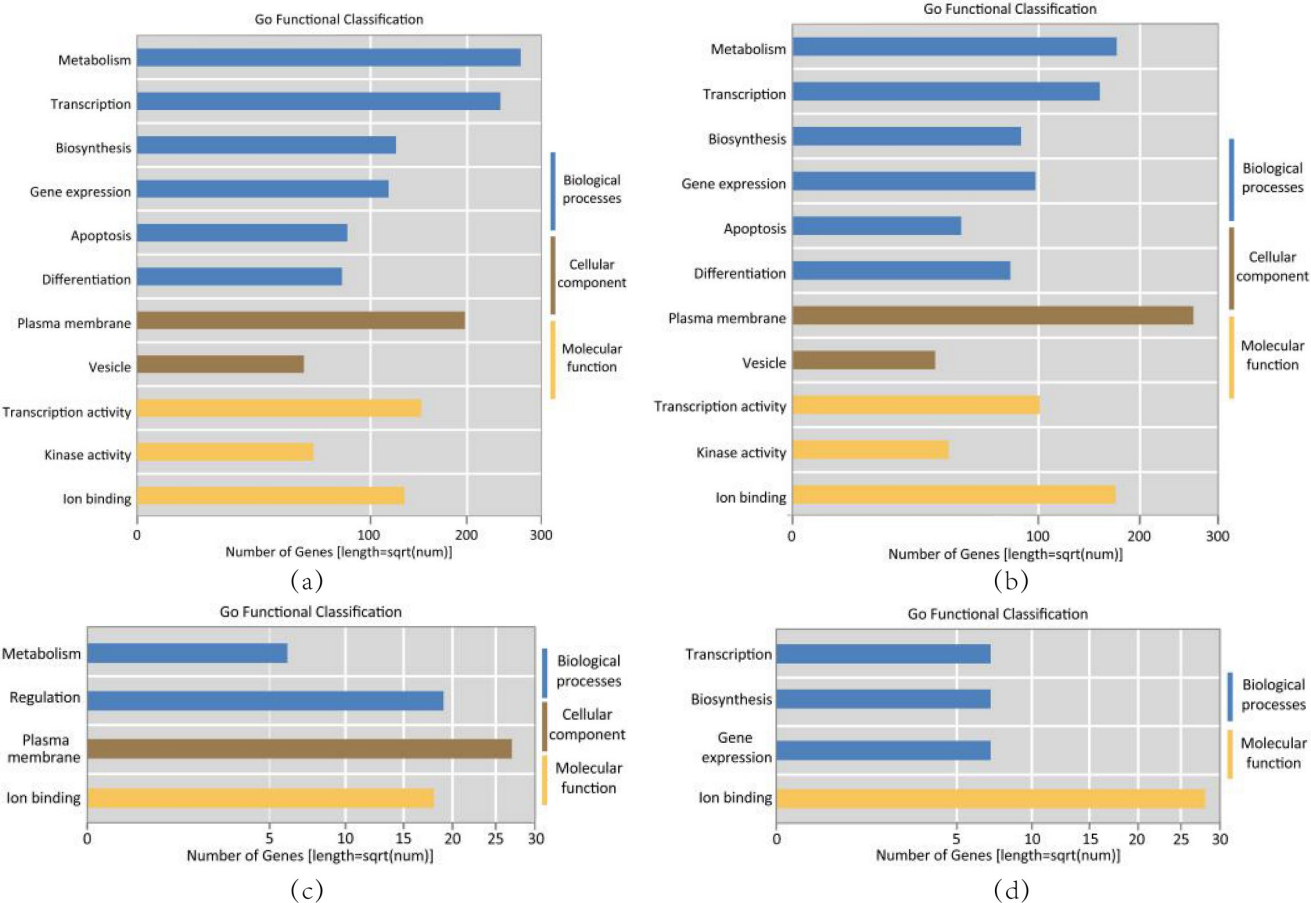
Biological features of miRNA combinations. (a) Sub 1; (b) Sub 2; (c) Sub 3; (d) Sub 4.

The GO enrichment analysis results with pvalue < 0.05 for Sub 1–4 (S6, S7, S8 and S9 Tables) are visualized in Fig 2. Sub 1 and Sub 2 were mainly enriched in metabolism, biosynthesis, apoptosis and differentiation in biological process (BP), plasma membrane and vesicle in cellular component (CC) and tanscription activity, kinase activity and ion binding in molecular function (MF). Sub 3 in metabolism and regulation in BP, plasma membrane in CC and ion binding in MF. Sub 4 in transcription, biosynthesis and gene expression in BP and ion binding in MF.

Part of the pathological factors inducing AF-VHD confirmed by the experiments include Ion channel change[25], inflammation[26–27], cellular fibrosis [28], cell proliferation and differentiation [29], Cardiovascular fibrosis[30], hormonal stimulation[31], energy metabolism[32] and protein matrix membrane system[33], etc. Some of these biological features above are related to the listed pathological factors inducing AF-VHD.

### Signaling pathways regulated by combinations of AF-VHD-specific miRNAs

12 significant signaling pathways (Pvalue<0.05) are enriched by KEGG to be related to AF-VHD (see Table 2). We found that a signaling pathway may be regulated by multiple combinations and a single combination of miRNAs may modulate multiple signaling pathways. For example, MAPK signaling pathway is regulated by Sub 1, Sub 2 and Sub 4, and Wnt signaling pathway by Sub 1 and Sub 2. Sub 1 and Sub 2 modulate 9 and 7 signaling pathways, respectively.

**Table 2:** The AF-VHD related signaling pathways regulated by combinatory miRNAs.

No.	The regulated signaling Pathways	Regulator	P
**1**	MAPK signaling pathway	Sub 1	0.0059
		Sub 2	0.0002
		Sub 4	0.0180
**2**	Wnt signaling pathway	Sub 1	0.0001
		Sub 2	0.0400
**3**	Focal adhesion	Sub 1	0.0008
		Sub 2	0.0011
**4**	ECM-receptor interaction	Sub 1	0.0220
		Sub 2	0.0140
**5**	Insulin signaling pathway	Sub 1	0.0025
		Sub 3	0.0220
**6**	p53 signaling pathway	Sub 1	0.0490
**7**	ErbB signaling pathway	Sub 1	0.0010
**8**	Phosphatidylinositol signaling system	Sub 1	0.0032
**9**	T cell receptor signaling pathway	Sub 1	0.0390
**10**	TGF-β signaling pathway	Sub 2	0.0490
**11**	Hypertrophic cardiomyopathy	Sub 2	0.0440
**12**	Jak-STAT signaling pathway	Sub 2	0.0460

## Discussion

### Potential roles of signaling pathways regulated by combinations of miRNAs in inducing AF-VHD

The regulation relationships between the combinatorial miRNAs and signaling pathways can be established via the target genes of this combination involved in the signaling pathways. The existing evidences can support that some of them play important roles in inducing AF-VHD.

**Fig 3:**
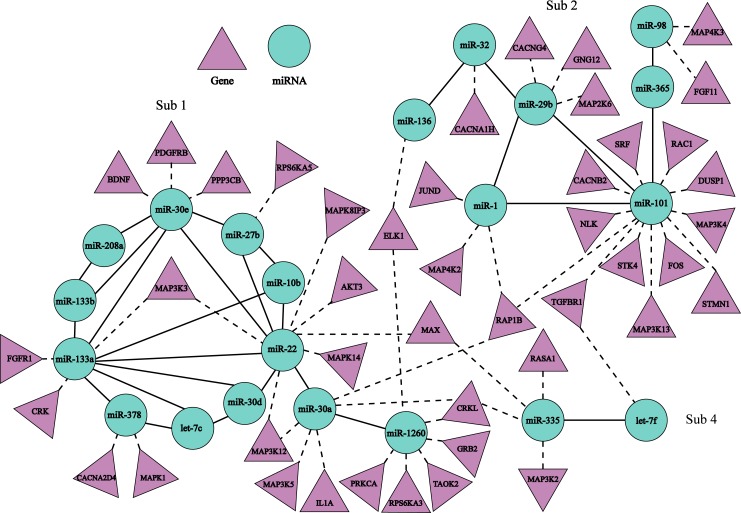
Regulation relationships among Sub 1, Sub 2 and Sub 4 and MAPK signaling pathway. A solid line indicates interaction relationship between miRNA pairs, and a dash line shows the regulation relationship between a miRNA and its target genes involved in MAPK signaling pathway.

The combinatorial Sub 1, Sub 2 and Sub 4 regulated MAPK signaling pathway. Fig 3 shows the regulations among Sub1, Sub2 and Sub4 and MAPK signaling pathway, constructed through the regulation relationship between miRNAs and their target genes in this pathway. The MAPK pathway is regulated by miRNA-1260, miRNA-133a, miRNA-22, miRNA-27b, miRNA-30e and miRNA-378 in the Sub1, miRNA-1, miRNA-101, miRNA-136, miRNA-296, miRNA-32 and miRNA-98 in Sub 2, and miRNA-335 and let-7f in Sub 4.

Conservative three grade kinase is the basic activation pattern of MAPK signaling transduction pathway. In other words, when the cells are stimulated, mitogen-activated protein (MAP) kinase kinase kinase (MKKK), MAP kinase kinase (MKK) and MAP kinase (MAPK) can be activated sequentially.

The activated MAPK protein, including Extracellular regulated protein kinase (ERK), C-Jun N-terminal kinase (JNK) and p38 MAPK, acts on the substrate to induce a series of physiological and pathological processes such as cell differentiation, apoptosis, inflammatory reaction and cytoskeletal changes.

The genes encoding MKKK include MAP3K12 and MAP3K (regulated by miR-30a in Sub 1), MAP3K5 and MAP3K3 (by miR-22 in Sub 1), MAP3K (by miR-133a in Sub 1), MAP3K13 and MAP3K4 (by miR-101 in Sub 2), and MAP3K2 (by miR-335 in Sub 4).

The genes encoding MKK have MAP2K6 (by miR-29b in Sub 2), and encoding MAPK proteins are MAPK14 (by miR-22 in Sub 1) and MAPK1 (by miR-378 in Sub 1). The substrate where p38 MAPK acts includes the genes FGFR1 (by miR-133a in Sub 1)[34], CACNA2D4 (by miR-378 in Sub 1) and CACNG4 (by miR-29b in Sub 2) [35] and TGFBR1 (by miR-101 in Sub 2 and let-7f in Sub 4) [36].

AF is characterized by structural remodeling, and its hallmark related to fibrosis, apoptosis and inflammatory reaction [37–41]. p38MAPK can regulate cardiomyocyte apoptosis and hypertrophy, inflammatory reaction and fibroblast activation [42–43]. Therefore, the differential expression of miRNAs in Sub 1, Sub 2 and Sub 4 between AF-VHD and VHD patients, which are involved in conservative three grade kinase reaction in MAPK signaling pathway, may affect conduction process of MAPK signaling through abnormal regulation of target genes and possibly result in structural cardiac remodeling.

**Fig 4:**
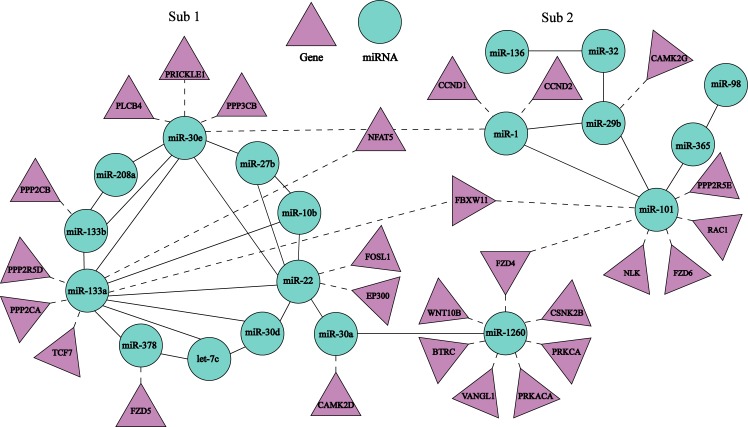
Regulation among Sub 1 and Sub 2 and Wnt signaling pathway. A solid line indicates interaction between two miRNAs, and a dash line shows the regulation relationship between a miRNA in a Sub and its target genes belonging to Wnt signaling pathway.

The combinatorial Sub 1 and Sub 2 regulate Wnt, Focal adhesion and ECM-receptor interaction signaling pathways, respectively (See Table 2). Fig 4 shows the regulation relationships among Sub 1 and Sub 2 and Wnt signaling pathway. The classic Wnt signaling pathway activates the transcription of downstream genes via β-Catenin. This pathway can be summarized as Wnt→FZD and LRP5/6→Dsh→Dissolution of the degradation complex of β-catenin→Accumulation and nuclei entry of β-catenin→TCF/LEF→ Transcription of genes such as CCND1 and CCND2 [44].

Fig 4 shows some miRNAs in Sub 1 and Sub 2 mediate the classic Wnt signaling pathway through the regulation of target genes. These miRNAs are miR-1260 in Sub 1 targeting WNT10B, miR-101 in Sub 2 targeting FDZ4 and FDZ6, miR-378 in Sub1 FDZ5, miR-133a in Sub 1 TCF7, and miR-1 in Sub 2 CCND1 and CCND2. Wherein, CCND1 and CCND2 can mediate proliferation, differentiation and apoptosis of cardiac muscle cells [45–46].

Additionally, the expression of gene NFAT5 is regulated by miR-30e (Sub1), miR-133a (Sub 1) and miR-1(Sub 2). In studying the relationships between P19CL6 cell line genes and differentiation of cardiac muscle cells, Adachi et al. [47] found NFAT5 involved in the classic Wnt signaling pathway mediates differentiation of cardiac muscle cells and remodels cardiac structure [40–41]. Therefore, Sub 1 and Sub 2 may regulate proliferation, differentiation and apoptosis of cardiac muscle cells via the classic Wnt signaling pathway, contributing to the remodeling of cardiac structure observed in AF.

The combinatorial Sub 1 regulates p53, ErbB, Phosphatidylinositol signaling system and T cell receptor signal pathways. p53 causes occurrence and maintenance of AF through the regulation of myocardial fibrosis and the atrial structural remodeling, which is one of AF characteristics [48]. Meanwhile, P53 has the following functions, such as inflammatory response, activating p53/NF-k, promoting myocardial fibrosis and cardiac structure remodeling through regulating apoptosis of cardiac muscle cells [49].

The combinatorial Sub 2 regulates TGF-β, Jak-STAT and Hypertrophic cardiomyopathy signal pathways. Hanna et al. found the expression of TGF-β in atrium of dogs with AF obviously increases in AF dog model. TGF-β leads to atrial fibrosis through promoting collagen synthesis, which causes occurrence and maintenance of atrial fibrillation [50].

In addition, Michelle et al. [51] found that the expression of TGF-β obviously increases in left ventricle and septum of rats with heart failure. TGF-β leads to fibrosis of cardiac muscle cell through synthesis of cell proliferation, adhesion, migration and extracellular matrix, which remodel cardiac structure.

### Comparison between the identified and existing AF-VHD-related DE miRNAs

The study of Cooply et al. [13] showed that there is no detectable effect of AF on miRNA expression in left atria (LA) tissue. Thus, our previous work only used the samples from right atrial myocardium (appendage) [14] to identify DE miRNAs associated with AF-VHD. However, other studies showed that there is detectable effect of AF on miRNA expression in LA tissue [52–54]. Thus, this study used the samples from left and right atrial myocardium (appendage) to identify the AF-VHD-related DE miRNAs.

In our previous and present work, APCA-Bootstrap method was respectively used to identify 47 DE miRNAs from two kinds of data under the condition of same parameters. There were 37 common and 10 different DE miRNAs between two results. The overlapping rate reached 78.8%. The different DE miRNAs between two works confirmed that there was detectable effect of chronic AF on miRNA expression in LA tissue.

Xiao et al. [12] found that 28 miRNAs are expressed differently in the VHD patients with AF compared with those without AF. Cooley et al. [13] identified 47 miRNAs showing differential expression between the AF-VHD and SR-VHD. Compared with the two previous results, there are 10 common miRNAs—hsa-let-7c, hsa-miR-133a, hsa-miR-133b, hsa-miR-181b, hsa-miR-21, hsa-miR-22, hsa-miR-30a, hsa-miR-30e, hsa-miR-335 and hsa-miR-378.

Reasons causing the low overlapping are as follows: (i) Tissue samples used by Xiao et al. and Cooley et al. are only from right atrial appendages, while our tissue samples from left and right atrial appendages. (ii) The DE miRNA identification method in the work is completely different with that used by Xiao et al. and Cooley et al. Inconsistent terminologies in DE miRNA identification produce different results. APCA-Bootstrap sampling algorithm identified DE miRNAs based on interaction relationships among miRNAs [14]. Xiao et al. and Cooley et al. used the traditional numerous feature selection methods [55] to identify DE miRNAs with significant differential expression values. It neglects those miRNAs with insignificant differential expression values but functioning with other miRNAs in disease development. (iii) The patients involved in three researches have individual differences.

### Differential co-expression network and AF-VHD-specific miRNAs

The work presented the new concept of differential co-expression network, with the proposal of its construction method. The AF-VHD-specific-miRNAs denoted by the nodes of this network have two types of differential properties—“difference between/within groups”. The former means that miRNAs are differentially expressed between the disease and control sample groups. The latter indicates the co-expression difference between two classes of samples. In most existing studies [12–14], the disease-specific miRNAs are usually identified only by the “difference between groups”. Since the co-expression within disease group has been confirmed to be different from that within control group [24], combining two types of differences is necessary to identify the disease-specific miRNAs. It can characterize pathophysiological aspects of a disease different from the control better.

## Conclusions

The work introduced the differential co-expression network to reveals two differences of “between groups” and “within groups” of a microarray expression profile with case-control samples. Based on constructed differential co-expression network, 32 AF-VHD-specific miRNAs and 5 combinations among them were identified, and some miRNAs were new findings. qRT-PCR experiment validated that part of identified miRNAs were differentially expressed. The biological characteristics of AF-VHD were highlighted through the enrichment analysis of target genes regulated by combinatorial AF-VHD-specific miRNAs. Twelve signaling pathways regulated by combinatorial AF-VHD-specific miRNAs were predicted to be possibly associated with AF-VHD. The potential roles of MAPK, Wnt, P53 and TGF-β signaling pathways in inducing AF-VHD were disclosed. These findings bring new insights for biomarkers, drug targets and miRNA combination regulation mechanism of AF-VHD as well as further biological experiments.

## Supporting information

S1 Table 47 DE miRMAs and the change of connection degree. (PDF)

S2 Table The normalized PCC among pairwise of 47 DE miRNAs. (PDF)

S3 Table Sequences of the primers used in the qRT-PCR validation. (PDF)

S4 Table qRT-PCR experiment results. (PDF)

S5 Table The target genes of combinatorial miRNAs. (PDF)

S6 Table The statistically enriched GO terms of Sub 1. (PDF)

S7 Table The statistically enriched GO terms of Sub 2. (PDF)

S8 Table The statistically enriched GO terms of Sub 3. (PDF)

S9 Table The statistically enriched GO terms of Sub 4. (PDF)

## Abbreviations

VHD: Valvular heart disease
MiRNA: microRNA
AF: Atrial fibrillation
SR: Sinus rhythm
AF-VHD: Valvular heart disease with atrial fibrillation
DE: Differentially expressed
mRNAs: Messenger RNAs
TPR: True positive rate
FDR: False positive rate
UTR: Untranslated region
RA: Right atrial
LA: Left atrial
UESTC: University of Electronic Science and Technology of China
APCA: Asymmetric principal component analysis
ROC: Receiver Operating Characteristic
AUC: Areas under the ROC curve
GO: Gene Ontology
KEGG: Kyoto Encyclopedia of Genes and Genomes
JNK: Jun N-terminal kinase
BP: Biological Process
CC: Cellular Component
MF: Molecular Function
PCC: Pearson correction coefficient
MAP: Mitogen-activated protein
MKKK: MAP kinase kinase kinase
MKK: MAP kinase kinase
MAPK: MAP kinase
