# Supplementary material for: Analysis of potential roles of combinatorial microRNA regulation in occurrence of valvular heart disease with atrial fibrillation based on computational evidences
Source: PLoS One. 2019 Sep 3;14(9):e0221900. doi: 10.1371/journal.pone.0221900 (PMC6719876; doi:10.1371/journal.pone.0221900)
Supplement: S1 Table — (PDF) [file pone.0221900.s001.pdf]

**S1 Table 47 DE miRMAs and the change of connection**

| No. | miRNA symbol | Change of connection degree |
|---|---|---|
| 1 | hsa-let-7b | 0 |
| **2** | **hsa-let-7c** | **3** |
| **3** | **hsa-let-7f** | **2** |
| **4** | **hsa-miR-1** | **3** |
| **5** | **hsa-miR-101** | **4** |
| 6 | hsa-miR-10a | 0 |
| **7** | **hsa-miR-10b** | **3** |
| **8** | **hsa-miR-1238** | **2** |
| **9** | **hsa-miR-1260** | **1** |
| 10 | hsa-miR-129-3p | 0 |
| **11** | **hsa-miR-133a** | **7** |
| **12** | **hsa-miR-133b** | **3** |
| 13 | hsa-miR-135a | 0 |
| **14** | **hsa-miR-136** | **2** |
| 15 | hsa-miR-143 | 0 |
| **16** | **hsa-miR-15b** | **1** |
| **17** | **hsa-miR-16** | **1** |
| **18** | **hsa-miR-181b** | **1** |
| 19 | hsa-miR-193a-3p | 0 |
| 20 | hsa-miR-200b | 0 |
| 21 | hsa-miR-203 | 0 |
| **22** | **hsa-miR-208a** | **2** |
| **23** | **hsa-miR-21** | **1** |
| **24** | **hsa-miR-214** | **1** |
| **25** | **hsa-miR-22** | **5** |
| 26 | hsa-miR-223 | 0 |
| 27 | hsa-miR-25 | 0 |
| **28** | **hsa-miR-27b** | **3** |
| **29** | **hsa-miR-29b** | **4** |
| 30 | hsa-miR-29c | 0 |
| **31** | **hsa-miR-30a** | **2** |
| **32** | **hsa-miR-30d** | **3** |
| **33** | **hsa-miR-30e** | **5** |
| **34** | **hsa-miR-32** | **1** |
| **35** | **hsa-miR-335** | **1** |
| **36** | **hsa-miR-361-3p** | **2** |
| **37** | **hsa-miR-362-5p** | **1** |
| **38** | **hsa-miR-365** | **2** |
| **39** | **hsa-miR-370** | **2** |
| **40** | **hsa-miR-378** | **2** |
| **41** | **hsa-miR-409-3p** | **1** |
| **42** | **hsa-miR-410** | **2** |
| 43 | hsa-miR-424 | 0 |
| 44 | hsa-miR-497 | 0 |
| 45 | hsa-miR-663 | 0 |
| 46 | hsa-miR-96 | 0 |
| **47** | **hsa-miR-98** | **1** |

**MiRNAs indicated in bold have non zero change of connection degree.**
