# Supplementary material for: Analysis of potential roles of combinatorial microRNA regulation in occurrence of valvular heart disease with atrial fibrillation based on computational evidences
Source: PLoS One. 2019 Sep 3;14(9):e0221900. doi: 10.1371/journal.pone.0221900 (PMC6719876; doi:10.1371/journal.pone.0221900)
Supplement: S3 Table — (PDF) [file pone.0221900.s003.pdf]

S3 Table Sequences of the primers used in the qRT-PCR validation.

Primer name	Primer sequence	Annealing temperature **(℃)**	Product length **(bp)**
U6 RT<br>U6: Forward<br>U6: Reverse	5'CGCTTCACGAATTTGCGTGTCAT3'<br>5'GCTTCGGCAGCACATATACTAAAAT 3'<br>5'CGCTTCACGAATTTGCGTGTCAT 3'	60	89
hsa-miR-32-5p RT<br><br>hsa-miR-32-5p: F<br>hsa-miR-32-5p: R	5'GTCGTATCCAGTGCGTGTCGTGGAGTCGGCAATTGCACTGGATACGACTGCAACT 3'<br>5' GGGGCTATTGCACATTACTA 3'<br>5' GTGCGTGTCGTGGAGTCG 3'	60	67
hsa-miR-98-5p RT<br><br>hsa-miR-98-5p: F<br>hsa-miR-98-5p: R	RT:5'GTCGTATCCAGTGCGTGTCGTGGAGTCGGCAATTGCACTGGATACGACAACAAT3'<br>5' GGGGGTGAGGTAGTAAGTTGT 3'<br>5' GTGCGTGTCGTGGAGTCG 3'	60	67
hsa-miR-30e-5p RT<br><br>hsa-miR-30e-5p: F<br>hsa-miR-30e-5p: R	5'GTCGTATCCAGTGCGTGTCGTGGAGTCGGCAATTGCACTGGATACGACCTTCCA 3'<br>5' GGGTGTAAACATCCTTGAC 3'<br>5' GTGCGTGTCGTGGAGTCG 3'	60	62
