# Supplementary material for: Analysis of potential roles of combinatorial microRNA regulation in occurrence of valvular heart disease with atrial fibrillation based on computational evidences
Source: PLoS One. 2019 Sep 3;14(9):e0221900. doi: 10.1371/journal.pone.0221900 (PMC6719876; doi:10.1371/journal.pone.0221900)
Supplement: S4 Table — (PDF) [file pone.0221900.s004.pdf]

S4 Table qRT-PCR experiment results

The experiment with U6 as a reference and sample 1 as a benchmark is corrected as follows:

Type	No. of Sample	U6	hsa-miR-32-5p	(hsa-miR-32-5p)Ct-(U6)Ct	[(hsa-miR-32-5p)Ct-(U6)Ct]sample-[(hsa-miR-32-5p)Ct-(U6)Ct]sample1	$2^{-\Delta\Delta CT}$	Average$2^{-\Delta\Delta CT}$
VHD	1	14.412	27.812	13.400	0.000	1.000	1.260
	2	14.581	27.153	12.572	-0.828	1.775	
	3	14.707	27.491	12.783	-0.617	1.533	
	4	14.449	27.765	13.316	-0.084	1.060	
	5	14.609	27.692	13.083	-0.317	1.246	
	6	14.753	28.238	13.485	0.085	0.943	
AF-VHD							0.622
	Ⅰ	14.603	28.103	13.500	0.100	0.933	
	Ⅱ	14.071	28.538	14.467	1.067	0.477	
	Ⅲ	14.523	28.627	14.104	0.704	0.614	
	Ⅳ	14.320	28.131	13.811	0.411	0.752	
	Ⅴ	14.558	28.852	14.294	0.894	0.538	
	Ⅵ	14.527	29.189	14.662	1.262	0.417	
	Ratio(AF-VHD/VHD)						0.494
	Pvalue						0.00221

Type	No. of Sample	U6	hsa-miR-98-5p	(hsa-miR-98-5p)Ct-(U6)Ct	[(hsa-miR-98-5p)Ct-(U6)Ct]sample-[(hsa-miR-98-5p)Ct-(U6)Ct]sample1	$2^{-\Delta\Delta CT}$	Average$2^{-\Delta\Delta CT}$
VHD	1	14.412	27.097	12.685	0.000	1.000	1.344
	2	14.581	26.403	11.822	-0.863	1.819	
	3	14.707	26.841	12.134	-0.551	1.465	
	4	14.449	26.770	12.322	-0.363	1.286	
	5	14.609	26.763	12.153	-0.532	1.446	
	6	14.753	27.366	12.613	-0.072	1.051	
AF-VHD							0.652
	Ⅰ	14.603	27.168	12.565	-0.120	1.087	
	Ⅱ	14.071	27.875	13.804	1.119	0.461	
	Ⅲ	14.523	28.148	13.625	0.940	0.521	
	Ⅳ	14.320	27.827	13.507	0.822	0.566	
	Ⅴ	14.558	27.516	12.958	0.273	0.827	
	Ⅵ	14.527	28.365	13.838	1.153	0.450	
	Ratio(AF-VHD/VHD)						0.485
	Pvalue						0.00157

Type	No. of Sample	U6	hsa-miR-30e-5p	(hsa-miR-30e-5p)Ct-(U6)Ct	[(hsa-miR-30e-5p)Ct-(U6)Ct]sample-[(hsa-miR-30e-5p)Ct-(U6)Ct]sample1	$2^{-\Delta\Delta CT}$	Average$2^{-\Delta\Delta CT}$
VHD	1	14.412	17.403	2.991	0.000	1.000	1.451
	2	14.581	16.998	2.417	-0.574	1.489	
	3	14.707	16.761	2.053	-0.938	1.915	
	4	14.449	16.964	2.515	-0.476	1.391	
	5	14.609	16.780	2.170	-0.821	1.766	
	6	14.753	17.551	2.798	-0.193	1.143	
AF-VHD							0.824
	Ⅰ	14.603	17.401	2.798	-0.193	1.143	
	Ⅱ	14.071	17.149	3.078	0.087	0.942	
	Ⅲ	14.523	18.643	4.120	1.129	0.457	
	Ⅳ	14.320	17.556	3.236	0.245	0.844	
	Ⅴ	14.558	17.716	3.158	0.167	0.891	
	Ⅵ	14.527	18.103	3.576	0.585	0.667	
	Ratio(AF-VHD/VHD)						0.568
	Pvalue						0.00467
