# Supplementary material for: Analysis of potential roles of combinatorial microRNA regulation in occurrence of valvular heart disease with atrial fibrillation based on computational evidences
Source: PLoS One. 2019 Sep 3;14(9):e0221900. doi: 10.1371/journal.pone.0221900 (PMC6719876; doi:10.1371/journal.pone.0221900)
Supplement: S7 Table — (PDF) [file pone.0221900.s007.pdf]

S7 Table The statistically enriched GO terms of Sub 2

GO	Style	Term	Pvalue	Gene count	The involved genes
Biological processes	Metabolic	positive regulation of RNA metabolic process	1.50E-10	155/424 (36.5%)	ARID1A,BCL11B,CTCF,ELK1,GATA6,KLF12,KLF13,KLF4,MKL2,MEIS2,NOTCH1,PKNOX1 etc
		positive regulation of macromolecule metabolic process	4.00E-10		KLF13,KLF4,MKL2,MEIS2,NDFIP1,NOTCH1,PKNOX1,SMAD7,SOX9,SMARCA1,SMARCA4 etc
		positive regulation of nitrogen compound metabolic process	2.40E-09		TBX3,TEAD3,ZIC3,ABLIM3,APP,ATXN1,BRPF1,CREB1,ESRRG,EYA1,GDF6,HAND2 etc
		negative regulation of nitrogen compound metabolic process	1.60E-05		CTCF,DNMT3A,KLF12,KLF4,MEIS2,NKRF,NDFIP1,SMAD7,SOX9,SMARCA4,TBX3,ATXN1 etc
		regulation of phosphorus metabolic process	2.40E-05		HMGCR,SMAD6,SMAD7,ADCY6,APP,APLP2,ATXN1,CAV3,CCND1,CCND2,CCNT2,CDK5R1 etc
		regulation of cellular protein metabolic process	3.70E-05		NDFIP1,SMAD6,SMAD7,TIMP3,APP,BOLL,CCND1,CCND2,CPEB1,CPEB2,DLC1 etc
		negative regulation of RNA metabolic process	7.20E-05		CTCF,DNMT3A,KLF12,KLF4,MEIS2,SMAD7,SOX9,SMARCA4,TBX3,BAZ2A,CBFA2T2 etc
		phosphorus metabolic process	1.80E-04		MARK1,NRK,SACM1L,SMAD7,SNRK,APP,CREB1,CAMK1D,CAMK2G,CAV2,CTTNBP2 etc
		phosphate metabolic process	1.80E-04		MARK1,NRK,SACM1L,SMAD7,SNRK,APP,CREB1,CAMK1D,CAMK2G,CAV2,CTTNBP2 etc
		negative regulation of macromolecule metabolic process	2.20E-04		CTCF,DNMT3A,KLF12,KLF4,MEIS2,NKRF,NDFIP1,SMAD6,SMAD7,SOX9,SMARCA4 etc
		positive regulation of protein metabolic process	2.10E-02		NDFIP1,SMAD7,BOLL,CCND1,CCND2,DLC1,GJA1,GCLC,GDF6,GHR,HDAC4,PPARGC1A etc
		regulation of glucose metabolic process	2.30E-02		DYRK2,HDAC4,IRS2,PPARGC1A,PGAM1,TGFBR1,UBE2D1,ZFP91
		regulation of carbohydrate metabolic process	3.30E-02		DYRK2,HDAC4,IRS2,PPARGC1A,PGAM1
	Transcription	regulation of transcription from RNA polymerase II promoter	1.20E-11	137/424 (32.3%)	BCL11B,DNMT3A,ELK1,GATA6,KLF12,KLF13,KLF4,MKL2,MEIS2,NOTCH1,PKNOX1,PKNOX2 etc
		positive regulation of transcription, DNA-dependent	1.10E-10		ABLIM3,APP,ATXN1,CREB1,ESRRG,HAND2,HDAC4,JUNB,MITF,NEUROD1,NFAT5,NR2F2 etc
		regulation of transcription	3.80E-10		HBP1,KHDRBS3,KLF12,KLF13,KLF4,MKL2,MEIS1,MEIS2,NKRF,NRK,NOTCH1,PKNOX1 etc
		positive regulation of transcription from RNA polymerase II promoter	4.20E-10		JUNB,MITF,NEUROD1,NFAT5,PAX3,PPARGC1A,RARB,RXRB,RUNX1,SRF,STAT6 etc
		positive regulation of transcription	7.60E-10		SP1,TBX3,TEAD3,ZIC3,ABLIM3,APP,ATXN1,BRPF1,CREB1,ESRRG,GDF6 etc
		regulation of transcription, DNA-dependent	2.20E-07		ARID1A,BCL11B,CTCF,DNMT3A,ELF5,ELK1,GATA6,KLF12,KLF13,KLF4,MKL2 etc
		transcription	5.90E-07		ARID1A,ARID4B,BCL11A,BCL11B,BCORL1,CTCF,ELF5,ELK1,EAF1,GATA6,HBP1 etc
		negative regulation of transcription	7.60E-06		CTCF,DNMT3A,KLF12,KLF4,MEIS2,NKRF,SMAD7,SOX9,SMARCA4,TBX3,ATXN1,BAZ2A etc
		negative regulation of transcription, DNA-dependent	5.50E-05		CTCF,DNMT3A,KLF12,KLF4,MEIS2,SMAD7,SOX9,SMARCA4,TBX3,BAZ2A,CBFA2T2,DR1 etc
		positive regulation of gene-specific transcription	3.70E-04		GATA6,NOTCH1,SMARCA1,SMARCA4,CREB1,HDAC4,NR2F2,PPARGC1A,SRF,TFE3,ETS1 etc
		regulation of gene-specific transcription	1.00E-03		GATA6,NOTCH1,SMAD7,SMARCA1,SMARCA4,TAL1,CREB1,HDAC4,NR2F2,PPARGC1A,SRF etc
		positive regulation of specific transcription from RNA polymerase II promo	1.90E-03		GATA6,NOTCH1,SMARCA4,CREB1,HDAC4,SRF,TFE3,ETS1
		regulation of specific transcription from RNA polymerase II promoter	2.70E-03		GATA6,NOTCH1,SMAD7,SMARCA4,TAL1,CREB1,HDAC4,SRF,TFE3,ETS1
		negative regulation of transcription from RNA polymerase II promoter	4.30E-03		DNMT3A,KLF12,MEIS2,SMAD7,SMARCA4,DR1,HDAC4,HOXB4,HIVEP1,JARID2,NR2F2 etc
	Biosynthetic	positive regulation of macromolecule biosynthetic process	4.00E-09	77/424 (18.1%)	ARID1A,BCL11B,CTCF,ELK1,GATA6,KLF12,KLF13,KLF4,MKL2,MEIS2,NOTCH1,PKNOX1 etc
		positive regulation of biosynthetic process	4.00E-09		BOLL,BRPF1,CREB1,DYRK2,ESRRG,GDF6,HAND2,HDAC4,IRS2,JUNB,MITF,NEUROD1 etc
		positive regulation of cellular biosynthetic process	6.70E-09		NFAT5,NR2F2,PAX3,PPARGC1A,RARB,RXRB,RUNX1,SRF,STAT6,STRN3,THRB,TFE3 etc
		negative regulation of cellular biosynthetic process	6.20E-06		DNMT3A,KLF12,KLF4,MEIS2,NKRF,SMAD7,SOX9,SMARCA4,TBX3,ATXN1,BAZ2A,CBFA2T2 etc
		negative regulation of macromolecule biosynthetic process	8.60E-06		EIF2C1,GCLC,HDAC4,HOXB4,HIVEP1,HIC2,ID4,IGFBP5,JARID2,NR2F2,PAX3 etc
		negative regulation of biosynthetic process	1.00E-05		NKRF,SMAD7,SOX9,SMARCA4,TBX3,ATXN1,BAZ2A,CBFA2T2,DR1,EED,EIF2C1,GCLC etc
	Gene expression	positive regulation of gene expression	2.00E-09	87/424 (20.5%)	ARID1A,BCL11B,CTCF,ELK1,GATA6,KLF12,KLF13,KLF4,MKL2,MEIS2,NOTCH1 etc
		negative regulation of gene expression	8.50E-06		MEIS2,NKRF,NDFIP1,SMAD7,SOX9,SMARCA4,TBX3,ATXN1,BAZ2A,CBFA2T2,DR1,EED etc
		posttranscriptional regulation of gene expression	1.10E-04		SMAD7,APP,BOLL,CPEB1,CPEB2,EIF2C1,EIF4E,EIF5,ETF1,IGFBP5,MTPN,NANOS1,PPARGC1A etc
		regulation of gene expression, epigenetic	2.70E-03		ARID1A,CTCF,DNMT3A,SMARCA4,SMARCD1,ATRX,BAZ2A,EIF2C1,FOS
	Apoptosis	positive regulation of apoptosis	2.90E-02	42/424 (9.9%)	BBC3,BCL2L11,NOTCH1,TIMP3,ALDH1A3,APP,CDK5R1,DLC1,DUSP1,DYRK2,ING3 etc
		regulation of apoptosis	2.50E-03		DUSP1,DYRK2,EYA1,GCLC,GHR,HSPE1,ING3,MITF,MAP2K6,NGFR,NEUROD1,PREX1,PIM1 etc
	Differentiation	negative regulation of cell differentiation	1.50E-05	70/424 (16.5%)	ABCA1,KLF4,NOTCH1,SMAD7,SOX9,TBX3,CCND1,CDK6,EZH2,HDAC4,ID4,NGFR,NRP1 etc
		neuron differentiation	4.90E-05		ID4,LIFR,MTPN,NGFR,NRP1,NRP2,OTX2,PAX3,PAFAH1B1,PBX3,RAC1,SRF,CELSR3 etc
		muscle cell differentiation	1.00E-04		ADAM12,GATA6,MKL2,APP,CACNA1H,CACNB2,CAV2,CAV3,MBNL1,MTPN,QKI,RARB etc
		cell morphogenesis involved in differentiation	2.40E-04		ATP2B2,BCL11B,NOTCH1,SOX9,APP,CREB1,CXCL12,CDK5R1,GAP43,LAMC1,NGFR,NRP1 etc
		cell morphogenesis involved in neuron differentiation	3.10E-04		ATP2B2,BCL11B,NOTCH1,APP,CREB1,CXCL12,CDK5R1,GAP43,NGFR,NRP1,NRP2 etc
		regulation of myeloid cell differentiation	3.10E-04		NDFIP1,TAL1,CDK6,MITF,PURB,RUNX1,TFE3,ETS1,MAFB,ZFP36
		striated muscle cell differentiation	1.70E-03		ADAM12,GATA6,APP,CACNA1H,CACNB2,CAV2,MTPN,RARB,RXRB,TTN
		cerebellar granule cell differentiation	5.60E-03		ATP2B2,MTPN,ULK1
		regulation of muscle cell differentiation	7.00E-03		NOTCH1,TBX3,CAV3,EZH2,HDAC4,SRF
		negative regulation of muscle cell differentiation	1.30E-02		NOTCH1,TBX3,EZH2,HDAC4
		regulation of striated muscle cell differentiation	1.70E-02		NOTCH1,TBX3,CAV3,EZH2,HDAC4
		regulation of erythrocyte differentiation	1.70E-02		TAL1,CDK6,ETS1,MAFB
		positive regulation of cell differentiation	2.80E-02		GATA6,NOTCH1,TAL1,TGIF2,JUNB,JUND,NGFR,NEUROD1,RUNX1,SRF,SOCS5 etc
		cardiac muscle cell differentiation	3.40E-02		GATA6,RARB,RXRB,TTN
Cellular component	Plasma membrane	plasma membrane	1.70E-03	237/336 （70.5%）	ADAM12,ADAM23,ABCA1,ATP2B2,ATP6V1B2,ATP1B1,ATP1B2,BCL2L11,BMF,CAP1 etc
		plasma membrane part	2.50E-03		CACNB2,CACNG4,CNR1,CAV2,CAV3,CLCN3,CTTNBP2,CDK5R1,CPEB1,DLC1,DCBLD2,DAG1 etc
		extrinsic to plasma membrane	9.70E-03		SMAD7,CAV2,GNB1,GNG12,GNG3,RAC1
		basal plasma membrane	3.00E-02		CAV2,CTTNBP2,ERBB2IP,MET
		internal side of plasma membrane	3.90E-02		RAB14,RAB39B,RAB40C,RAP2C,RAPH1,AP3S1,CACNB2,CAV2,CDK5R1,DMD etc
	Vesicle	vesicle membrane	4.30E-05	30/336 (8.9%)	SEC24C,CACNG4,CAMK2G,CAV2,CLCN3,CTTNBP2,EPN2,GJA1,ITPR1,PHLDA1,SCAMP1,SLC32A1 etc
		cytoplasmic vesicle membrane	7.30E-05		SEC24C,CACNG4,CAMK2G,CAV2,CTTNBP2,EPN2,GJA1,ITPR1,PHLDA1,SCAMP1,SLC32A1 etc
		cytoplasmic vesicle part	1.20E-04		CAMK2G,CAV2,CTTNBP2,EPN2,FGA,GJA1,ITPR1,PHLDA1,SCAMP1,SLC32A1,SYT1 etc
		membrane-bounded vesicle	2.10E-03		ABCA1,ATP6V1B2,DNAJC5,NOTCH1,RAB14,SEC24C,AP3S1,APP,CACNG4,CAMK2G,CAV2 etc
		cytoplasmic membrane-bounded vesicle	2.70E-03		RAB14,SEC24C,AP3S1,APP,CACNG4,CAMK2G,CAV2,CTTNBP2,EPN2,FGA,GJA1,ITPR1 etc
		cytoplasmic vesicle	3.10E-03		RAB14,ARHGAP21,SEC24C,AP3S1,APP,CACNG4,CAMK2G,CAV2,CLCN3,CTTNBP2,ENTPD7 etc
		vesicle	5.50E-03		CACNG4,CAMK2G,CAV2,CLCN3,CTTNBP2,ENTPD7,EPN2,FGA,GJA1,ITPR1,MYRIP etc
		transport vesicle	2.90E-02		RAB14,SEC24C,AP3S1,CAV2,SYT1,VAMP2
		coated vesicle membrane	4.20E-02		SEC24C,EPN2,SCAMP1,SLC32A1,SYT1,VAMP2
		clathrin coated vesicle membrane	5.00E-02		EPN2,SCAMP1,SLC32A1,SYT1,VAMP2
Molecular function	Transcription activity	transcription regulator activity	1.90E-09	90/408 (22.0%)	AEBP2,ARID1A,BCL11A,CTCF,ELF5,ELK1,GATA6,HBP1,KLF12,KLF13,KLF4 etc
		RNA polymerase II transcription factor activity	5.00E-08		PKNOX1,SOX12,SOX9,SP1,SP2,TBX3,TEAD3,CREB1,HOXC10,JUNB,JUND etc
		transcription factor activity	3.10E-07		CTCF,ELF5,ELK1,GATA6,KLF12,KLF4,MEIS1,MEIS2,NRK,NOTCH1,PKNOX1,PKNOX2 etc
		transcription repressor activity	7.20E-06		CTCF,KLF12,KLF4,MEIS2,NKRF,TBX3,ATXN1,CBFA2T2,DR1,EED,EZH2,HDAC4 etc
		transcription activator activity	9.50E-05		ARID1A,CTCF,ELK1,GATA6,KLF4,MKL2,NOTCH1,SOX9,SMARCA4,SMARCD1,SP1,TEAD3 etc
		specific RNA polymerase II transcription factor activity	3.40E-04		MEIS2,PKNOX1,SOX9,CREB1,NR2F2,RXRB,SRF,FOS
		RNA polymerase II transcription factor activity, enhancer binding	7.10E-03		GATA6,PKNOX1,CREB1,MITF,PAX3,TFAP4
		transcription corepressor activity	1.60E-02		CBFA2T2,DR1,ID4,JUNB,NR2F2,THRB
		transcription cofactor activity	2.00E-02		AEBP2,BCL11A,CTCF,KLF12,MKL2,MEIS2,SMARCD1,CREB1,CBFA2T2,DR1,HAND2
	Kinase activity	protein kinase activity	3.60E-04	36/408 (8.8%)	MARK1,NRK,SNRK,CAMK1D,CAMK2G,CAV2,CTTNBP2,CCND1,CDK5R1,CDK6,CDK9,CRIM1,DYRK1A etc
		protein serine/threonine kinase activity	4.90E-04		CAMK1D,CAMK2G,CDK5R1,CDK9,DYRK1A,DYRK2,ICK,MAP2K6,MAP3K13,MAP3K4,MAP4K2,MAP4K3 etc
		protein tyrosine kinase activity	3.60E-02		CAV2,CTTNBP2,CRIM1,DYRK1A,DYRK2,MET,MAP2K6,NRP1,NRP2,STK4,TTN
	Ion binding	metal ion binding	3.60E-02	154/408 (37.7%)	BPNT1,ADAM12,ADAM23,ADAMTS18,ADAMTS6,ADAMTS9,ADAMTSL3,AEBP2,ATP2B2,ATP1B1,ATP1B2 etc
		ion binding	3.90E-02		NOX4,NOTCH1,PAPPA,PHF15,PHF3,REV3L,SEC24C,SNRK,SP1,SP2,TAF15,ZIC3,ABLIM3 etc
		magnesium ion binding	4.70E-02		ATP2B2,MARK1,SNRK,ACSL4,ADCY6,DYRK2,EYA1,EYA4,GCLC,ICK,MAP3K13,MAP3K4,MYLK etc
