# Supplementary material for: Analysis of potential roles of combinatorial microRNA regulation in occurrence of valvular heart disease with atrial fibrillation based on computational evidences
Source: PLoS One. 2019 Sep 3;14(9):e0221900. doi: 10.1371/journal.pone.0221900 (PMC6719876; doi:10.1371/journal.pone.0221900)
Supplement: S6 Table — (PDF) [file pone.0221900.s006.pdf]

S6 Table The statistically enriched GO terms of Sub 1

GO	Style	Term	Pvalue	Gene count	The involved genes
Biological processes	Metabolic	regulation of RNA metabolic process	1.20E-10	270/662 (40.7%)	SLA2,TBX4,TAL1,TBPL1,TSC22D4,WDTC1,ATXN7L3,BAZ2A,ABL1,CALCR,CDX2 etc
		positive regulation of RNA metabolic process	3.20E-09		E2F3,ELK1,FOSL1,KLF12,MKL2,MEIS2,POU4F2,PRDM16,RBM15,SATB2,SOX4,SMARCD1 etc
		positive regulation of macromolecule metabolic process	2.70E-08		NFYA,PPARA,PPARG,PPARGC1A,PPARGC1B,PSMD7,PRKCA,RARB,RUNX1,SREBF2,SUPT4H1 etc
		negative regulation of RNA metabolic process	4.40E-07		E2F6,JAZF1,JDP2,KLF12,MXD4,MEIS2,PHF21A,POU4F2,PRDM1,PRDM16,PAWR etc
		positive regulation of nucleobase, nucleoside, nucleotide and nucleic acid metabolic p	7.70E-07		ARID1A,EP300,E2F3,ELK1,FOSL1,KITLG,KLF12,MKL2,MEIS2,POU4F2,PRDM16,RBM15 etc
		negative regulation of macromolecule metabolic process	1.00E-06		ATP8B1,BCL2L1,BNIP3L,JAZF1,JDP2,KLF12,MXD4,MEIS2,PHF21A,POU4F2,PRDM1 etc
		positive regulation of nitrogen compound metabolic process	2.10E-06		ARID1A,EP300,E2F3,ELK1,FOSL1,KITLG,KLF12,MKL2,MEIS2,POU4F2,PRDM16 etc
		phosphorus metabolic process	1.50E-05		BCL2,EPHA7,EPHB2,GAB1,NRK,PAN3,CDK16,PDIK1L,SACM1L,SBF1,SNRK,SPEG,TAOK2,TYRO3 etc
		phosphate metabolic process	1.50E-05		CSF1R,CDK5R1,DCLK1,EYA2,FGFR1,FLT1,HIPK1,IPPK,INSR,ICK,MAPK1,MAPK14,MAPK8IP3 etc
		negative regulation of nucleobase, nucleoside, nucleotide and nucleic acid metabolic p	2.30E-05		ATP8B1,E2F6,JAZF1,JDP2,KLF12,MXD4,MEIS2,PHF21A,POU4F2,PRDM1,PRDM16 etc
		negative regulation of nitrogen compound metabolic process	3.30E-05		ATP8B1,E2F6,JAZF1,JDP2,KLF12,MXD4,MEIS2,PHF21A,POU4F2,PRDM1,PRDM16 etc
		regulation of cellular protein metabolic process	1.00E-03		ADAM9,BCL2,CD28,EP300,GAB1,KITLG,RB1CC1,TIMP3,BTRC,BOLL,CASC3,CPEB1 etc
		regulation of cellular carbohydrate metabolic process	9.70E-03		HDAC4,INSR,IRS1,IGF1,NR3C1,PPARGC1A,PLEK
		regulation of carbohydrate metabolic process	1.10E-02		HDAC4,INSR,IRS1,IGF1,NR3C1,PPARGC1A,PLEK
		regulation of phosphorus metabolic process	1.40E-02		ADAM9,BCL2,GAB1,KITLG,RB1CC1,SH3BP5,TAOK2,ADCY6,CAMKK2,CAV3,CHRM1 etc
		regulation of phosphate metabolic process	1.40E-02		ADAM9,BCL2,GAB1,KITLG,RB1CC1,SH3BP5,TAOK2,ADCY6,CAV3,CHRM1,CCNG1 etc
		positive regulation of protein metabolic process	1.60E-02		ADAM9,BCL2,CD28,EP300,KITLG,RB1CC1,BTRC,BOLL,EIF5A,GJA1,HDAC4
		positive regulation of cellular protein metabolic process	2.00E-02		ADAM9,BCL2,CD28,EP300,KITLG,RB1CC1,BTRC,BOLL,EIF5A2,FURIN,INSR,IGF1 etc
		regulation of glucose metabolic process	2.60E-02		HDAC4,INSR,IRS1,IGF1,NR3C1,PPARGC1A
		collagen metabolic process	4.60E-02		EPHB2,ARHGDIA,MBP,SEMA3A,SEMA4F
	Transcription	regulation of transcription	8.90E-15	242/662 (36.5%)	AFF3,AFF4,ARID1A,ARID3A,ARID3B,ATP8B1,BCL11A,BCL9L,BTG1,BCORL1,BACH1,BACH2 etc
		transcription	1.70E-13		KLF12,MNT,MXD4,MKL2,MAX,MEIS1,PHF21A,POU3F1,PRDM1,PRDM16,PRDM4,PAWR etc
		regulation of transcription from RNA polymerase II promoter	1.10E-11		EP300,E2F6,ELF2,ELK1,FOSL1,JAZF1,JDP2,KLF12,MXD4,MKL2,MEIS2 etc
		regulation of transcription, DNA-dependent	1.80E-10		ETV1,FOXA1,FOXD1,FOXG1,FOXL2,FOXM1,FOXP4,GTF2H1,HLF,HDAC4,HOXA1,HOXA11 etc
		positive regulation of transcription, DNA-dependent	2.30E-09		POU4F2,PRDM16,RBM15,SATB2,SOX4,SMARCD1,SP1,ATXN7L3,CDX2,CLOCK,CBFB,CSRNP2 etc
		positive regulation of transcription from RNA polymerase II promoter	4.30E-08		IGF1,JUNB,MAML1,MTF1,MAPK14,NEUROD1,NEUROG2,NPAS4,NFIB,NFAT5,NRIP1 etc
		positive regulation of transcription	1.30E-07		ARID1A,EP300,E2F3,ELK1,FOSL1,KLF12,MKL2,MEIS2,POU4F2,PRDM16,RBM15 etc
		negative regulation of transcription from RNA polymerase II promoter	2.60E-07		E2F6,JAZF1,JDP2,KLF12,MXD4,MEIS2,PHF21A,POU4F2,PRDM1,PRDM16,PAWR etc
		negative regulation of transcription, DNA-dependent	2.80E-07		RBM15,SATB1,SATB2,SLA2,WDTC1,BAZ2A,CDX2,CBX5,PRDM1,PRDM16,PAWR etc
		negative regulation of transcription	1.20E-06		ATP8B1,E2F6,JAZF1,JDP2,KLF12,MXD4,MEIS2,PHF21A,POU4F2,PRDM1,PRDM16 etc
		transcription from RNA polymerase II promoter	3.00E-05		AFF4,E2F3,FOSL1,MNT,MAX,PRDM4,TARDBP,TBPL1,ASH1L,CDX2,CBFB,CCNT2 etc
		regulation of gene-specific transcription	5.90E-03		RBM15,TAL1,WDTC1,HDAC4,IGF1,NCOR2,NR2F2,NR5A2,PPARA,PPARG,PPARGC1A
		chromatin-mediated maintenance of transcription	1.30E-02		ARID1A,SMARCD1 ,MLL4
		regulation of specific transcription from RNA polymerase II promoter	4.00E-02		RBM15,TAL1,WDTC1,HDAC4,NCOR2,PPARA,PPARG,SIRT1,TFE3,ETS1
		negative regulation of specific transcription from RNA polymerase II promoter	4.80E-02		RBM15,WDTC1,HDAC4,NCOR2,PPARA,PPARG
	Biosynthetic	positive regulation of macromolecule biosynthetic process	5.70E-09	123/662 (18.5%)	ARID1A,CD28,EP300,E2F3,ELK1,FOSL1,KITLG,KLF12,MKL2,MEIS2,POU4F2,PRDM16 etc
		positive regulation of cellular biosynthetic process	3.50E-08		ATXN7L3,BOLL,CAMKK2,CDX2,CLOCK,CBFB,CSRNP2,EPC1,ESRRG,EIF5A2,FOXA1 etc
		positive regulation of biosynthetic process	6.20E-08		EIF5A2,FOXA1,FOXL2,GTF2H1,HDAC4,HOXA1,HOXB9,INSR,IRS1,IGF1,IL1A,JUNB etc
		negative regulation of macromolecule biosynthetic process	1.40E-06		ATP8B1,E2F6,JAZF1,JDP2,KLF12,MXD4,MEIS2,PHF21A,POU4F2,PRDM1,PRDM16,PAWR etc
		negative regulation of biosynthetic process	2.50E-06		ATP8B1,E2F6,JAZF1,JDP2,KLF12,MXD4,MEIS2,PHF21A,POU4F2,PRDM1,PRDM16,PAWR etc
		negative regulation of cellular biosynthetic process	6.30E-06		ATP8B1,E2F6,AZF1,JDP2,KLF12,MXD4,MEIS2,PHF21A,POU4F2,PRDM1,JARID2 etc
		RNA biosynthetic process	6.40E-04		AFF4,E2F3,FOSL1,MNT,MAX,PRDM4,TARDBP,TBPL1,ASH1L,CDX2,CBFB,CCNT2,ELL2 etc
		negative regulation of receptor biosynthetic process	2.20E-02		SLC1A2,SLC1A3,SLC7A10
	Gene expression	positive regulation of gene expression	2.80E-08	116/662 (17.5%)	MAPK1,MAPK14,MLL4,NEUROD1,NEUROG2,NPAS4,NFIB,NFAT5,NRIP1,NR2F2,NR4A2 etc
		negative regulation of gene expression	4.30E-08		EIF2C1,EIF2C3,XPO5,FOXA1,FOXP4,HDAC4,HIC2,INSR,JARID2,KDM6B,MECP2,NCOR2 etc
		posttranscriptional regulation of gene expression	6.60E-04		MKL2,SPEG,ACTA1,CAV3,DMD,EGR3,FOXL2,FXR1,HSPG2,IGF1,MAPK14,MEF2D,NR2F2 etc
		positive regulation of gene expression, epigenetic	4.30E-02		ARID1A,SMARCD1,MLL4
	Apoptosis	regulation of apoptosis	7.00E-05	81/662 (12.2%)	CDK5R1,CDKN1A,CRTAM,ESR1,FOXL2,FURIN,HIPK1,HSPA5,IGF1,IGF2R,IL1A etc
		regulation of programmed cell death	9.40E-05		ADAM9,BCL2,BTG1,BBC3,BCL2L1,BCL2L2,BNIP3L,CD3G,DPF2,EPHA7,FOSL1,GCH1 etc
		regulation of cell death	1.00E-04		MAP3K5,MCL1,NET1,NEUROD1,NR3C1,NR4A2,PTEN,PREX1,PGP,KCNMA1,PHB etc
		positive regulation of apoptosis	1.10E-03		FOSL1,GCH1,PAWR,ARHGEF12,ARHGEF3,SOX4,TIMP3,ABL1,CUL2,CUL3,CDK5R1 etc
		positive regulation of programmed cell death	1.30E-03		BBC3,BCL2L1,BNIP3L,DPF2,EPHA7,FOSL1,PAWR,ARHGEF12,ARHGEF3,SOX4,TIMP3 etc
		positive regulation of cell death	1.40E-03		BCL2,BBC3,BCL2L1,DPF2,EPHA7,FOSL1,GCH1,ARHGEF12,ARHGEF3,SOX4 etc
		induction of apoptosis	7.70E-03		BBC3,BNIP3L,DPF2,GCH1,ARHGEF12,ARHGEF3,TIMP3,ABL1,C16orf5,CUL2,CUL3 etc
		induction of programmed cell death	8.00E-03		CUL3,CDKN1A,CRTAM,FOXL2,HIPK1,MAPK1,MAP3K5,NET1,PTEN etc
		regulation of neuron apoptosis	1.20E-02		ADAM9,BCL2,BCL2L1,EPHA7,BDNF,CDK5R1,ESR1,FURIN,NR3C1,NR4A2 etc
		apoptosis	4.30E-02		BCL2,BBC3,BCL2L1,BCL2L2,BNIP3L,DPF2,DDIT4,EP300,PAWR,ARHGEF12,ARHGEF3
	Differentiation	cell morphogenesis involved in neuron differentiation	3.70E-06	77/662 (11.6%)	NDEL1,LPPR4,PRKCA,PTPN11,PTPRZ1,SEMA3A,SEMA3B,SEMA4F,SLC1A3,UNC5C,ULK2 etc
		cell morphogenesis involved in differentiation	8.50E-06		NRXN3,NEUROG2,NRP2,NR4A2,NDEL1,LPPR4,PRKCA,PTPN11,PTPRZ1,SEMA3A,SEMA3B etc
		regulation of myeloid cell differentiation	5.40E-04		KITLG,PRDM16,RBM15,TAL1,MAPK14,PURB,RUNX1,HMGB3,SMAP1,TFE3,TOB2 etc
		neuron differentiation	8.40E-04		POU4F2,BDNF,CNTN4,CUX1,CDK5R1,DMD,EMX1,EFNB1,FGFR1,FOXA1,FOXG1 etc
		central nervous system neuron differentiation	2.90E-03		EPHB2,DCLK1,FOXG1,HOXA1,NEUROG2,NR4A2,NDEL1,PRKCA
		fat cell differentiation	3.90E-03		PRDM16,ADRB1,INSR,PPARG,PPARGC1A,RGS2,SIRT1,SLC2A4,SOCS1
		positive regulation of cell differentiation	4.20E-03		BCL2,BTG1,CD83,EPHB2,POU4F2,ARHGDIA,TAL1,BDNF,CDX2,FOXA1,FOXG1,HOXA11
		negative regulation of cell differentiation	4.60E-03		EPHB2,PRDM16,RBM15,ARHGDIA,BDNF,CDX2,CNTN4,FOXG1,HDAC4,IGF1 etc
		brown fat cell differentiation	5.40E-03		PRDM16,ADRB1,PPARG,PPARGC1A,RGS2,SLC2A4
		trophectodermal cell differentiation	6.40E-03		SP1,SP3 ,CDX2,CUL3,JUNB
		regulation of neuron differentiation	1.30E-02		GAB1,WDTC1,ADCY6,GRB2,INSR,IRS1,JUNB,PRKACA,PTPN1,PTPN11
		positive regulation of erythrocyte differentiation	1.40E-02		TAL1,MAPK14,SMAP1,ETS1
		positive regulation of myeloid cell differentiation	2.10E-02		KITLG,TAL1,MAPK14,RUNX1,SMAP1,ETS1
Cellular component	Plasma membrane	plasma membrane part	3.00E-06	197/540 （36.4%)	ADAM9,ATP2A2,ATP2B1,ATP6AP2,ATP8B1,BCL2L1,CD28,CD3G etc
		plasma membrane	3.00E-04		ADAM9,ARFIP2,AAK1,ATP2A2,ATP2B1,ATP6AP2,ATP8B1,BCL2L1,CD28,CD3G etc
		integral to plasma membrane	6.20E-04		ADRB1,ACCN1,CALCR,CCR5,CHRM1,CSF1R,DCBLD2,DCLK1,EFNA3,EFNB1,FGFR1 etc
		intrinsic to plasma membrane	7.40E-04		FLT1,FZD5,GABRA4,GABRA5,GJA1,GRIN3A,GRM5,HAS3,INSR,IRS1,IGF2R etc
		internal side of plasma membrane	1.50E-02		BCL2L1,CADPS,FRMPD1,RAB15,RAB38,RAB5B,RAP2B,RAP2C,RAPH1,RHEBL1,WDFY3 etc
		external side of plasma membrane	2.80E-02		ADAM9,ATP6AP2,CD28,CD83,CD8A,CCR5,ITGB3,MMP15,MMP16,LPPR4,KCNMA1,SLC2A4 etc
	Vesicle	cytoplasmic vesicle	3.90E-05	51/540 (9.4%)	CADPS,DENND1A,GCH1,N4BP3,RAB11A,RAB38,RAB5B,RACGAP1,SLA2,TAOK2 etc
		vesicle	5.80E-05		CADPS,DENND1A,GCH1,N4BP3,RAB11A,RAB38,RAB5B,RACGAP1,SLA2,TAOK2 etc
		membrane-bounded vesicle	9.90E-04		DENND1A,RAB11A,RAB38,RAB5B,RACGAP1,SLA2,TAOK2,AP2A1,AP4E1,BECN1 etc
		cytoplasmic membrane-bounded vesicle	1.00E-03		BDNF,BIN1,CALCR,CAMK2D,CALU,CLTA,SLA2,TAOK2,AP2A1,AP4E1,BECN1 etc
		transport vesicle	1.70E-03		RAB11A,AP2A1,CLTA,FURIN,IGF2R,NCALD,SLC2A4,SYT1,VAMP2
		trans-Golgi network transport vesicle	2.90E-03		AP2A1,CLTA,FURIN,IGF2R,NCALD,SLC2A4
		Golgi-associated vesicle	5.30E-03		AP2A1,AP4E1,CLTA,FURIN,GJA1,IGF2R,NCALD,SLC2A4
		clathrin coated vesicle membrane	6.50E-03		DENND1A,AP2A1,CLTA,NCALD,SCAMP1,SV2A,SYT1,VAMP2
		clathrin-coated vesicle	9.90E-03		DENND1A,AP2A1,BIN1,CLTA,FURIN,IGF2R,NCALD,SCAMP1,SLC2A4,SORT1,SLC2A4,SV2A,SYT1
		vesicle membrane	1.10E-02		DENND1A,AP2A1,AP4E1,CAMK2D,CLTA,GJA1,GRB2,ITGB3,NCALD,SCAMP1,SLC2A4,SV2A,SYT1,VAMP2
		coated vesicle membrane	1.10E-02		DENND1A,AP2A1,AP4E1,CLTA,NCALD,SCAMP1,SV2A,SYT1,VAMP2
		coated vesicle	1.70E-02		DENND1A,AP2A1,AP4E1,CAMK2D,CLTA,GJA1,GRB2,ITGB3,NCALD,SCAMP1,SLC2A4 etc
		transport vesicle membrane	2.20E-02		AP2A1,CLTA,NCALD,SYT1,VAMP2
		cytoplasmic vesicle part	2.70E-02		DENND1A,AP2A1,AP4E1,CAMK2D,CLTA,GJA1,IGF1,ITGB3,NCALD,SCAMP1,SV2A etc
		Golgi-associated vesicle membrane	3.20E-02		AP2A1,AP4E1,CLTA,GJA1,NCALD
		cytoplasmic vesicle membrane	3.30E-02		DENND1A,AP2A1,AP4E1,CAMK2D,CLTA,GJA1,ITGB3,NCALD,SCAMP1,SV2A,SYT1,VAMP2
Molecular function	Transcription activity	transcription regulator activity	2.70E-15	148/639 (23.1%)	AFF4、ARID1A、ARID3A、BCL11A、BCL2、BTG1、BACH1、BACH2、EP300、E2F3 etc
		transcription factor activity	8.30E-11		AFF4、ARID3A、BACH1、BACH2、EP300、E2F3、E2F6、ELF2、ELK1、FOSL1、GATAD2B etc
		transcription activator activity	7.30E-07		ARID1A、BCL2、EP300、E2F3、ELF2、ELK1、FOSL1、MNT、MKL2、MAX、POU3F1、PRDM16
		transcription repressor activity	5.00E-06		BCL11A、E2F6、ELF2、JAZF1、JDP2、KLF12、MNT、MEIS2、PRDM16、PAWR、SETD8、SP3 etc
		RNA polymerase II transcription factor activity	2.30E-05		MEIS1、MEIS2、PRDM4、SOX12、TBPL1、ASH1L、CBFB、CUX1、ELL2、FOXM1、GTF2H1 etc
		transcription cofactor activity	9.50E-05		BTG1、EP300、E2F6、JAZF1、KLF12、MNT、MXD4、MKL2、MAX、MEIS2、PRDM16、PAWR etc
		transcription corepressor activity	2.50E-04		BCL11A、E2F6、JAZF1、KLF12、MNT、MXD4、MEIS2、PAWR、SETD8、CDX2、JUNB、MECP2、NCOR2 etc
		transcription coactivator activity	3.30E-02		EP300、MNT、MKL2、MAX、PRDM16、SMARCD1、SMARCD2、TBPL1、ATXN7L3、CBFB etc
	Kinase activity	transmembrane receptor protein tyrosine kinase activity	8.40E-05	57/662 (8.6%)	EPHA7、EPHB2、TYRO3、CSF1R、EFNA3、FGFR1、FLT1、INSR、IRS1、IGF2R、NRP2、PDGFRB etc
		protein kinase activity	9.70E-05		AKAP9、AAK1、EPHA7、EPHB2、NRK、PAN3、CDK16、PDIK1L、SNRK、TAOK2、TYRO3 etc
		protein serine/threonine kinase activity	6.80E-03		AKAP9、AAK1、NRK、CDK16、PDIK1L、SNRK、SPEG、TAOK2、CAMK2D、CAMKK2、CSNK1G1、CSNK1G3
		protein tyrosine kinase activity	7.20E-03		EPHA7、EPHB2、TYRO3、ABL1、CAMKK2、CSF1R、EFNA3、FGFR1、FLT1 etc
		protein kinase inhibitor activity	7.40E-03		SH3BP5、CDKN1A、PRKACA、SFN、SOCS1、SOCS3、TRIB2
		kinase inhibitor activity	8.40E-03		SH3BP5、CDKN1A、PRKACA、SFN、SOCS1、SOCS3、TRIB2
		protein kinase regulator activity	1.70E-02		SH3BP5、CDK5R1、CDKN1A、CSNK2B、PRKACA、SFN、SOCS1、SOCS3、TRIB2、ERBB3
		kinase regulator activity	3.80E-02		SH3BP5、CDK5R1、CDKN1A、CSNK2B、PRKACA、SFN、SOCS1、SOCS3、TRIB2、ERBB3
	Ion binding	zinc ion binding	3.90E-02	131/639 (20.5%)	NT5E、ADAM19、ADAM22、ADAM9、ADAMTS3、ADAMTS9、BCL11A、DPF2、EP300、FBXL19 etc
