# Supplementary material for: Analysis of potential roles of combinatorial microRNA regulation in occurrence of valvular heart disease with atrial fibrillation based on computational evidences
Source: PLoS One. 2019 Sep 3;14(9):e0221900. doi: 10.1371/journal.pone.0221900 (PMC6719876; doi:10.1371/journal.pone.0221900)
Supplement: S8 Table — (PDF) [file pone.0221900.s008.pdf]

S8 Table The statistically enriched GO terms of Sub 3

GO	Style	Term	Pvalue	Gene count	The involved genes
Biological processes	Metabolic	positive regulation of nitrogen compound metabolic pro	2.40E-02	6/36 (16.6%)	CLOCK,ESRRA,GRIN1,MAP2K1,NFATC3,ZNF423
		positive regulation of RNA metabolic process	3.50E-02		CLOCK,ESRRA,GRIN1,MAP2K1,NFATC3
	Regulation	homophilic cell adhesion	3.20E-11	19/36 (52.7%)	PCDHA11,PCDHA12,PCDHA10, PCDHA13, PCDHAC1, PCDHAC2,PCDHA1, PCDHA4,PCDHA2,PCDHA3,PCDHA5,PCDHA7,PCDHA6, PCDHA8,PCDHA9
		cell-cell adhesion	1.20E-09		NLGN1,PCDHA11,PCDHA12,CDHA10, PCDHA13, PCDHAC1, PCDHAC2,PCDHA1, PCDHA4,PCDHA2,PCDHA3,PCDHA5,PCDHA7,PCDHA6, PCDHA8,PCDHA9
		cell adhesion	8.80E-07		WISP1,NLGN1,PCDHA11,PCDHA12,PCDHA10, PCDHA13, PCDHAC1, PCDHAC2,PCDHA1, PCDHA4,PCDHA2,PCDHA3,PCDHA5,PCDHA7,PCDHA6, PCDHA8,PCDHA9
		biological adhesion	8.90E-07		NLGN1,PCDHA11,PCDHA12,PCDHA10, PCDHA13, PCDHAC1, PCDHAC2,PCDHA1, PCDHA4,PCDHA2,PCDHA3,PCDHA5,PCDHA7,PCDHA6, PCDHA8,PCDHA9
Cellular component	Plasma membrane	integral to plasma membrane	8.80E-05	27/33 (81.8%)	GRIN1,NLGN1,KCNJ2,PCDHA11,PCDHA10, PCDHA13, PCDHAC1, PCDHAC2,PCDHA1, PCDHA4,PCDHA2,PCDHA3,PCDHA5,PCDHA7,PCDHA6, PCDHA8,SLC6A4
		intrinsic to plasma membrane	1.10E-04		GRIN1,NLGN1,KCNJ2,PCDHA11,PCDHA10, PCDHA13, PCDHAC1, PCDHAC2,PCDHA1, PCDHA4,PCDHA2,PCDHA3,PCDHA5,PCDHA7,PCDHA6, PCDHA8,SLC6A4
		plasma membrane	4.40E-03		CACNB1, GRIN1, MAP2K1, NLGN1, KCNJ2, PCDHA11, PCDHA12, PCDHA10, PCDHA13, PCDHAC1, PCDHAC2, PCDHA1, PCDHA4, PCDHA2, PCDHA3, PCDHA5 etc
		plasma membrane part	5.00E-03		CACNB1, GRIN1, NLGN1, KCNJ2, PCDHA11, PCDHA10, PCDHA13, PCDHAC1, PCDHAC2, PCDHA1, PCDHA4, PCDHA2, PCDHA3, PCDHA5, PCDHA7, PCDHA6, etc
Molecular function	Ion binding	calcium ion binding	7.10E-06	18/39 (46.1%)	ASPH, CACNB1, GRIN1, PCDHA11, PCDHA12, PCDHA10, PCDHA13, PCDHAC1, PCDHAC2, PCDHA1, PCDHA4, PCDHA2, PCDHA3, PCDHA5, PCDHA7 etc
