# Supplementary material for: Analysis of potential roles of combinatorial microRNA regulation in occurrence of valvular heart disease with atrial fibrillation based on computational evidences
Source: PLoS One. 2019 Sep 3;14(9):e0221900. doi: 10.1371/journal.pone.0221900 (PMC6719876; doi:10.1371/journal.pone.0221900)
Supplement: S9 Table — (PDF) [file pone.0221900.s009.pdf]

S9 Table The statistically enriched GO terms of Sub 4

GO	Style	Term	Pvalue	Gene count	The involved genes
Biological processes	Transcription	positive regulation of transcription	2.00E-02	7/53 (13.2%)	BCL11B,ISL1,SMARCAD1,SP1,HAND1,NR4A3,TGFBR1
		positive regulation of gene expression	2.30E-02		BCL11B,ISL1,SMARCAD1,SP1,HAND1,NR4A3,TGFBR1
	Biosynthetic	positive regulation of macromolecule biosynthetic process	3.90E-02	7/53 (13.2%)	BCL11B,ISL1,SMARCAD1,SP1,HAND1,NR4A3,TGFBR1
		positive regulation of cellular biosynthetic process	4.70E-02		BCL11B,ISL1,SMARCAD1,SP1,HAND1,NR4A3,TGFBR1
		positive regulation of biosynthetic process	4.90E-02		BCL11B,ISL1,SMARCAD1,SP1,HAND1,NR4A3,TGFBR1
	Gene expression	positive regulation of transcription	2.00E-02	7/53 (13.2%)	BCL11B,ISL1,SMARCAD1,SP1,HAND1,NR4A3,TGFBR1
Molecular function	Ion binding	metal ion binding	5.70E-03	28/56 (46.1%)	ADAM19,BCL11B,CHFR,EEF2K,NR4A3,ZBTB5,RNF141, etc
		cation binding	6.60E-03		ADAM19,BCL11B,CHFR,EEF2K,NR4A3,ZBTB5,RNF141, etc
		ion binding	8.20E-03		ADAM19,BCL11B,CHFR,EEF2K,NR4A3,ZBTB5,RNF141, etc
