# Supplementary material for: Analysis of potential roles of combinatorial microRNA regulation in occurrence of valvular heart disease with atrial fibrillation based on computational evidences
Source: PLoS One. 2019 Sep 3;14(9):e0221900. doi: 10.1371/journal.pone.0221900 (PMC6719876; doi:10.1371/journal.pone.0221900)
Supplement: S5 Table — (PDF) [file pone.0221900.s005.pdf]

S5 Table The target genes of combinatorial miRNAs

combination	MicroRNA target prediction	
	MicroRNA	Gene
	hsa-let-7c	PPP1R15B
		BACH1
		E2F6
		AKAP6
		ACTA1
		BTBD3
		THBS1
	hsa-miR-10b	NCOR2
		DAZAP1
		ZNF367
		DOCK11
		FXR2
		ELAVL2
		ARIH2
		HOXA1
		NR4A3
		CECR6
		GRIN3A
		MTMR3
		LPHN1
		BCL2L2
	hsa-miR-27b	RPS6KA5
		C5orf13
		TXN2
		CD28
		PPARG
		CCNG1
		ADD3
		NCOA7
		VAV3
		POM121
		KITLG
		SLC38A4
		PHB
		KIAA1737
		CDK5R1
		NHS
		XPO1
		ST14
		HOXC11
		INSR
		ANTXR2
		PLEKHJ1
		SEMA3B

		ADAM22
		STIM2
		BAZ2B
		RAP2C
		UBE2J1
		YTHDF3
		FAM43A
		ZNF644
		STK39
		ZNRF1
		CA10
		CADPS
		CHD7
		GOLGA4
		GABRA5
		ADAM19
		TRPM7
		ZDHHC21
		USP48
		USP37
		BECN1
		RAP1B
		PAWR
		COL13A1
		FOXD1
		CALU
		PTPN2
		ELL2
		SOX4
		IL1A
		SOCS3
		INSIG2
		SOCS1
		ARID1A
		ERG
		CAMTA1
		CALCR
		JPH4
		PSMD7
		ADAMTS9
		NR5A2
		MAP3K5
		MEIS2
		SNAI1
		EML4
		DLL4
		FBXL17

		hsa-miR-30a	SNAI2
			ZDHHC17
			GLDC
			SORCS3
			YWHAZ
			SATB2
			GALNT3
			SEMA6D
			HIC2
			AP2A1
			DDIT4
			RUNX1
			PDE4D
			DMD
			MAP3K12
			SLC38A2
			CLOCK
			CAMK2D
			SLC7A10
			NRIP1
			POU4F2
			CARS
			GRM5
			PDCL
			SLC4A7
			FBXL20
			ACTR1A
			SGCB
			BNC1
			SATB1
			STC1
			NAGPA
			MYBL2
			HOXA11
			EPHB2
			EAF1
			RAI14
			SMARCD2
			ICK
			FLJ36031
			NRK
			NFIB
			IGF2R
			TIMP3
			NEDD4L
			IDH1
			LPP

		DEXI
		TSGA14
		EIF5A2
		UNC5D
		STK35
		EFNA3
		ADRB1
		CCPG1
		USP44
		CPEB4
		ITGB3
		CSNK1G1
		EIF2C1
		TNXB
		COPS7B
		MBD6
		THBS2
		IER2
		ESRRG
		TP53INP1
		ATP2A2
		CECR6
		RASL12
		ARID3A
		MEF2D
		SLC6A15
		SLC36A1
		SCAMP1
		NAV3
		CAMKK2
		ELMO1
		FXR1
		NR2F2
		MKL2
		DDX46
		MAN1B1
		PITX1
		BACH1
		NR3C2
		ARHGEF3
		PDP2
		RASGEF1A
		CRKL
		EGR3
		PLXNA1
		KLF12
		DIO2

			PLCG1
			FOXA1
			BCL2
			JUNB
			ROD1
			BNC2
			NCALD
			SLC1A2
			MECP2
			BCL11A
			HIPK1
			FOXP4
			SIAH2
			B4GALT5
			RASAL2
			CHFR
			FRMD4A
			CORO2A
			AP3M1
			NAV1
			SNX27
			DCP2
			BACH2
		hsa-miR-30d	SUPT3H
			KCTD5
			HSPA5
			SBF1
		hsa-miR-133b	PPP2CB
			PDIA4
			FOXG1
			NPAS4
			NEUROD1
			SPEG
			C5orf41
			TOB2
			C16orf72
			H3F3B
			CD3G
			SLC2A1
			PAPOLG
			CHIT1
			RRP1B
			SFT2D3
			ARRDC2
			XPNPEP3
			RPL23A
			SOX7

	hsa-miR-378	MREG
		PDIK1L
		HDAC4
		CRLF3
		PAPOLA
		CACNA2D4
		TLK2
		PAPPA
		XPO5
		MAPK1
		DLAT
		ZNF507
		TRIL
		SAR1A
		RIT1
		FZD5
		KPNA6
		ANKRD52
		MKL2
		MEF2D
		RNF41
		SLC30A8
		SARM1
		MAPK1IP1L
		DDX3X
		IGF1
		CALN1
		WDR37
		SEC63
		MNT
		NUFIP2
		SLC38A1
		LDLRAD2
		TFCP2L1
		SETD8
		CBX5
		OTUD7B
		DBT
		ELFN2
		MTF1
		AAK1
		MED13L
		RNF41
		PTBP2
		BZRAP1
		UGT1A10
		UGT1A8

		UGT1A7
		UGT1A6
		UGT1A5
		UGT1A9
		UGT1A4
		UGT1A1
		UGT1A3
		CSNK2B
		UNC13C
		LARP1B
		NT5C2
		MSTO1
		BTBD11
		EIF2C1
		CSRNP2
		SEMA3A
		RELT
		C17orf59
		WIBG
		GPKOW
		XKR6
		CC2D1B
		C14orf1
		KCNIP1
		RNF146
		LHFPL1
		PRDM4
		TIMM17A
		TDRD9
		ZNF629
		CUX1
		C2orf84
		WNT10B
		PTPRU
		INPP5A
		AMBRA1
		HM13
		ZNF594
		GJA5
		GLP1R
		NRXN2
		HMX2
		C1orf63
		FXR2
		PPP1R8
		ZHX2
		CALU

		TARDBP
		BSDC1
		EMX1
		ZBTB6
		C22orf29
		POU3F1
		SORT1
		CYTIP
		UBE2E1
		LRRTM4
		WDR82
		AK2
		STMN4
		PGM5
		PIP5K1A
		SMOC1
		RACGAP1
		ZNF544
		DBN1
		TAF15
		DNAJB1
		NUMA1
		CHRM1
		TAOK2
		TCF20
		FOXM1
		IPPK
		MLL4
		KDM4A
		N4BP3
		C6orf132
		TBX4
		FAM120C
		DCBLD2
		SRRM2
		ACCN1
		SLA2
		GREM2
		ELK1
		GRRP1
		UBTF
		SP6
		TAL1
		WTAP
		ERLIN1
		ZNF48
		NPAS4

			AFF3
			PTPN11
			ATP2B1
			ASH1L
			KCTD12
			FAM109B
			GNPNAT1
			CDK16
			PLEK
			STAG2
			FXR1
			POGZ
			ETS2
			RNF121
			BBC3
			SCD
			PTGFRN
			LAMC2
			MLL2
			ZNF385A
			ZNF763
			RNF123
			KIAA1161
			SYNM
			ZNF781
			ETV1
			SLC16A3
			LDOC1L
			ZNF740
		hsa-miR-1260	SREBF2
			CD8A
			KLHDC7A
			SH3BP5
			PI4KB
			MMP16
			TMEM104
			TNPO3
			DIXDC1
			VAMP1
			ZNF814
			C6orf195
			FLT1
			DCLK1
			DLG2
			ETS1
			SMYD4
			MGLL

Sub 1		SEMA4F
		HOXB5
		SUPT4H1
		ZNF559
		ZNF541
		JPH2
		CCDC117
		ATXN7L3
		HOXC10
		FBXL19
		NCOR2
		C12orf53
		XPR1
		HAS3
		SURF4
		EFNB1
		TSC22D4
		RAP2B
		LIMD2
		KNCN
		SMARCD1
		PER1
		ST3GAL3
		HOXB13
		COL1A1
		RAPGEFL1
		ZNF428
		RAB42
		ELAVL3
		GATAD2B
		PLCXD3
		HOXB9
		RPS6KA3
		DENND1A
		ATP8B1
		KDM6B
		BTRC
		SUFU
		PDXK
		AFF4
		MEX3A
		CD83
		CCDC93
		CCR5
		SCRN1
		SF3B3
		GAB1

		DPYSL3
		ELL2
		SFN
		PRKACA
		GLT25D2
		WWC3
		KPNA6
		RBMS2
		PTPN3
		ZFHX2
		ANP32A
		USP27X
		CENPP
		SLC2A4
		GRB2
		DHRS7B
		CRKL
		SLC31A1
		CUL2
		ZBTB46
		IGF2BP1
		AP3M1
		NFIX
		CNOT6L
		LRRC55
		NFIB
		LHFPL4
		SLC30A5
		FZD4
		PIGK
		SOX12
		GAB2
		EIF2S1
		ZNF266
		PGP
		NPTX1
		MXD4
		NEUROG2
		SIK2
		MYO5B
		B3GALNT2
		SLA
		CNKSR2
		SESTD1
		ANTXR2
		MAPRE2
		ZFP3

		MMP15
		GJB4
		ZNF780B
		PHF21A
		WIZ
		FASN
		ZNF260
		AGPS
		DCAF7
		ENAH
		INO80D
		GABRA4
		VANGL1
		KLHL24
		C10orf114
		PRKCA
		CCDC97
		TNRC6B
		SLC7A8
		TFE3
		QKI
		PPP2R5D
		BCL2L1
		ARFIP2
		ADCY6
		FGFR1
		ALDH5A1
		EIF2C1
		ZNF436
		SENP1
		SYNPO2L
		SLC6A6
		NIPA1
		FOXP4
		C16orf5
		KPNA6
		TTYH3
		FBXL19
		HIC2
		JDP2
		C10orf46
		NFAT5
		WHSC2
		SP1
		NLGN2
		SNX1
		LHFP

		SLC6A1
		UBA2
		NUP153
		SGPP1
		CLTA
		PTPRZ1
		VPS54
		EIF4A1
		SEC61B
		SMARCD1
		PPP2CA
		SOX4
		SUMO1
		PTPRO
		TIMM17A
		GPM6A
		SH3GL2
		SNRK
		SEPHS2
		BAZ2A
		FTL
		SP3
		TFAP2D
		PTBP2
		PFN2
		BNIP3L
		TBPL1
		RARB
		RAP2C
		GCH1
		EPHA7
		ADCYAP1
	hsa-miR-133a	PEX5L
		MEIS2
		SACM1L
		VAMP2
		SYT1
		AKAP9
		CRTAM
		CSNK1G3
		ARHGAP12
		KCNMA1
		ATP6AP2
		TMOD3
		FBXW11
		BTBD3
		SOLH

		DOLPP1
		LASP1
		TRHDE
		FBXL2
		MTMR4
		RB1CC1
		RAVER1
		SV2A
		ANKRD12
		LRRC7
		FOXL2
		MAML1
		SLC4A1
		MCL1
		PAN3
		GRM5
		USP32
		PTPRD
		MEIS1
		GDI2
		ELAVL1
		YES1
		TCF7
		SUPT16H
		BCORL1
		RAPH1
		ARHGDIA
		JAZF1
		CRK
		CASC3
		RCE1
		TRAM2
		ELF2
		NDRG1
		XPO4
		MLLT3
		BCL2L2
		PRDM16
		USP6
		PPFIA3
		MAP3K3
		GABARAPL1
		NRIP3
		VAPB
		SESN1
		CD47
		PFKFB3

		PREX1
		WASF1
		H3F3B
		RBM15
		AKT3
		NET1
		ARPC5
		GATM
		FAM49B
		SEPHS1
		STK39
		CPEB1
		CHD9
		BOLL
		OGN
		RGS2
		SIRT1
		STAG2
		POGK
		CHD7
		MAX
		RAB5B
		LRRC1
		PTEN
		IL13RA1
		TRIB2
		SATB2
		CDX2
		HOXA4
		LAMC1
		TRUB1
		RSBN1
		FMNL2
		FNBP4
		CSF1R
		RFXANK
		CALCR
		TYRO3
		EMILIN3
		COPS7B
		DNM3
		MAPK14
		FBXL19
		EP300
		ERBB3
		CAV3
		TP53INP1

		hsa-miR-22	LIN7C
			PPP1R15B
			AMOT
			PTPN9
			SMAP1
			ODF1
			ESR1
			ARFIP2
			FBXW7
			ZFYVE9
			JARID2
			NDEL1
			MAP3K12
			NUDT4
			SNRK
			BCL9L
			BCL9
			MTHFD2
			KIAA1274
			NR3C1
			FBN2
			DNAJB5
			SV2A
			DPP10
			EPC1
			FOSL1
			BTG1
			WDTC1
			DPF2
			IPO7
			PTPN1
			CUL3
			DPYSL3
			PLEKHA6
			SLC2A1
			SLC16A2
			SLC1A3
			RAPGEFL1
			RIMS4
			PHF13
			GALNT4
			TAGLN
			DGKI
			CALM3
			WDFY3
			HSPG2
			SLC9A5

		IL17RD
		PURB
		BIN1
		NFYA
		MXD4
		LGALS1
		ASB6
		CDKN1A
		ARID3B
		KIAA0355
		USP37
		TLK2
		KCNK10
		TTYH3
		ZCCHC8
		ARHGEF12
		MAP3K3
		PPP1R16B
		SLC17A5
		MECP2
		FURIN
		MBP
		MAPK8IP3
		USP46
		HDAC4
		ULK2
		C20orf112
		SP1
		PPARA
		PTPN13
		WDR7
		UBE2V2
		LRRC17
		PAPD4
		LPPR4
		NT5E
		PRDM1
		NAALADL2
		HLF
		DOLPP1
		CCNT2
		ATP2B1
		ELOVL5
		SCN3A
		NR4A2
		UBE2I
		LGI1

		PAPOLA
		CAPZA1
		ADAMTS3
		PFN2
		CHST2
		NRXN3
		C9orf72
		FAP
		RAB15
		PCDH17
		UNC5C
		RAB32
		BNIP3L
		RAB11A
		ADAM9
		AP4E1
		FRMPD1
		SLC5A11
		GATM
		EIF2C3
		IRS1
		CTNND2
		MMD
		NRP2
		PON2
		PAPOLB
		NHLH2
		RAB38
		LPHN3
		DHX40
		PPARGC1B
		PTP4A1
		PDCD10
	hsa-miR-30e	PPARGC1A
		PGM1
		CBFB
		C20orf108
		GFPT2
		RHEBL1
		CALB2
		E2F3
		SSBP2
		GJA1
		ABL1
		SIRT1
		KIAA0355
		PLS1

		ESPN
		GTF2H1
		CNTN4
		SEMA6B
		RGL1
		RNF122
		TAB3
		PRICKLE1
		ITPK1
		PPP3CB
		KCTD3
		NRBF2
		KCTD7
		CAPN5
		SEC61A2
		JDP2
		VAT1
		TBPL1
		TRPS1
		EYA2
		TUBGCP3
		PDGFRB
		PGGT1B
		NFAT5
		BDNF
		OSBPL8
		SOX12
		GPT2
		PLCB4
		IRX4
		GOT2
		PLCXD3
		SLC6A6
		COTL1
		PHF13
		HMGB3
		SLC39A11
		PTPN21
		GJA1
		DHX15
		MET
		HS3ST3B1
		CXorf23
		ANXA4
		NRP1
		GNPDA2

			EIF4E
			CLCN3
			FBXO33
			CPEB1
			MYLK
			JARID2
			BPNT1
			H3F3B
			OSBPL7
			NAP1L5
			RARB
			TBC1D15
			MEOX2
			SNAP25
			SLC25A22
			HMGCR
			ADAM12
			RNF138
			BCL11A
			HOXB4
			UBE4A
			MEIS1
			KCTD10
			VAMP2
			ZNF146
			CORO1C
			EIF1AX
			BSCL2
			OTX2
			FRAS1
			GDF6
			SP2
			GAS2L1
			BOLL
			CORO1B
			STAG2
			C20orf112
			JUND
			SERP1
			SNAI2
			RAP1B
			LIN7C
		hsa-miR-1	QKI
			ARF3
			GIT1
			EYA4
			CAP1

		EAF1
		KCTD13
		SEC63
		PREX1
		SMARCA4
		CDK9
		SYT1
		CRIM1
		PAFAH1B1
		KLF4
		HDAC4
		KLF13
		BLCAP
		NFAT5
		GCLC
		ARHGAP21
		DNAJC5
		EIF2C1
		EML4
		CCND1
		DLC1
		CELSR3
		SLC38A2
		CCND2
		ATP6V1B2
		RIMS4
		SLC31A1
		PGAM1
		MAP4K2
		MUM1L1
		TRPS1
		NGFR
		GNPNAT1
		TFE3
		IGFBP5
		HSPE1
		MTX1
		NRP2
		PIM1
		LETM1
		CDK6
		HIC2
		THRB
		ZFP91
		TBX3
		SLC25A1
		STC1

		FLRT3
		GLTSCR1
		ZNF207
		MYCN
		ASPN
		CDYL
		ZFHX4
		ATP1B1
		PAPOLG
		RAP1B
		EYA1
		UBE2D1
		MYRIP
		MAP3K13
		EZH2
		NOVA1
		CAMTA1
		SLC12A2
		PRKAA1
		ZNF532
		NUDT11
		FOS
		SEL1L
		ZCCHC2
		NEUROD1
		CPEB3
		SMARCA1
		C20orf20
		APP
		SOCS5
		ERBB2IP
		MBNL1
		CDH11
		ARID1A
		UBE2D2
		PPFIA1
		GNB1
		MTMR2
		KHDRBS3
		ANKRD17
		ID4
		REV3L
		RANBP9
		KCNH7
		NLK
		BTBD3
		FZD6

		NDFIP1
		FBN2
		GJA1
		DUSP1
		DR1
		RAP2C
		AEBP2
		ADAMTSL3
		RAB39B
		NRK
		COL10A1
		STMN1
		BICD2
		PBX3
		CDH5
		DCBLD2
		STAG2
		DNMT3A
		RAB1A
		SULT4A1
		AP3S1
		STIM2
		SOX9
		ZFX
		RAB14
		SACM1L
		PACRG
		BCL9
		SOCS2
		LRRN1
		EED
		CACNB2
		ING3
		KIAA1409
		CTTNBP2
		CPEB2
		FZD4
		ATRX
		USP38
		EMP1
		ACSL4
		RBBP7
		GPR85
		FGA
		CDK5R1
		MET
		RXRB

			PCDH8
			FBXW11
			TOP1
			PTGS2
			MARK1
			USP47
			CBFA2T2
			HDGFRP3
			TGFBR1
			DAG1
			ATP5G2
			IPO8
			CAV3
			LRRFIP2
			SLC1A1
			ELF5
			PUM2
			SMARCA4
			TERF2
			RASD2
			RNF38
			ICK
			CTCF
			TULP4
		hsa-miR-101	PHLDA1
			SLC19A2
			MKL2
			SMARCD1
			MITF
			FLRT2
			LRRC4
			SORCS3
			TRIB1
			MEIS2
			TAL1
			DDIT4
			STRN3
			KIAA0182
			DYRK1A
			RAC1
			TLK2
			POGZ
			PURB
			CCNJ
			ZNF238
			PNRC1
			KIAA1244

	**Sub 2**	G3BP2
		PANK1
		BZW1
		BAZ2A
		ARID4B
		PTPN9
		PHF3
		SPRED1
		PDE4D
		BRPF1
		PCDH18
		NR2F2
		EPN2
		QKI
		GDF10
		PPP2R5E
		ATXN1
		MYO1E
		TEAD3
		ACCN2
		MAP3K4
		RBL2
		ZNF24
		SCAMP1
		ALDH1A3
		RNF139
		KPNA3
		TFAP4
		SHANK2
		RAPH1
		ADCY6
		CDC42EP3
		STAT6
		OGT
		FMR1
		CCNT2
		ABLIM3
		ATP1B2
		RREB1
		DMD
		TTN
		CPEB1
		ATP2B2
		CXCL12
		AMMECR1
		TGIF2
		PPP2R2A

		PDE4A
		SLC4A4
		KBTBD2
		ABCA1
		CXorf23
		STARD8
		SLC25A3
		JUNB
		PIM1
		N4BP1
		C6orf62
		EPB41L1
		SGPL1
		SLC30A7
		MEF2D
		APLP2
		MGRN1
		RGL1
		RAPGEF5
		C13orf23
		HAPLN1
		SNN
		NPTX1
		IGFBP5
		EIF5
		PKNOX1
		RNF44
		DYRK2
		SRF
		TIMP3
		ETS1
		APPBP2
		RUNX1
		TBC1D14
		PPP1R16B
		NDRG4
		STK4
		BBC3
		DGCR2
		DLC1
		ANTXR2
		SIK2
		ARHGAP26
		GRM1
		NANOS1
		LIFR
		MSI1

		ANKRD12
		KLF12
	hsa-miR-136	MTMR4
		RGS4
		MTPN
		MDM1
		GLT6D1
		TRPC4AP
		RAP2C
		INO80
		ETF1
		HOXC10
		GDF6
		PPARGC1A
		RNF139
		ZIC3
		ESRRG
		NOVA1
		MOSPD1
		EXTL3
		CPEB2
		PSME4
		SLC7A3
		SNRK
		ZNF436
		NRK
		GNG3
		IGFBP5
		ELK1
		USP37
		PURB
		PLCXD3
		COL3A1
		HBP1
		COL15A1
		COL4A1
		ZFP36L1
		CAV2
		BCL11A
		C5orf13
		COL4A5
		ADAMTS9
		PPIC
		CCNT2
		COL2A1
		BLMH
		ADAMTS6

	hsa-miR-29b	RARB
		COL7A1
		PAIP2
		NKRF
		PPM1D
		LAMC1
		CAMK2G
		MAFB
		GPX7
		HAS3
		C13orf23
		TFAP2C
		MFAP2
		BMF
		FBXW9
		ADAMTS7
		CAMK1D
		CNR1
		ARMC8
		TP53INP2
		ZBTB5
		HDAC4
		EML5
		PRR3
		BCORL1
		ADAMTS18
		PLEKHA1
		SS18L1
		SCHIP1
		PER3
		HPCAL4
		FRAS1
		IMPDH1
		C1QTNF6
		PKNOX2
		GNG12
		ZFP36
		SOX12
		PPP1R3D
		ENTPD7
		ZFP91
		NAV1
		MAP2K6
		BCL11B
		INA
		RAB40C
		CACNG4

		ITGA6
		GPR85
		SP1
		SLC16A2
		RERE
		CBX6
		RNF122
		AQP4
		FCHSD1
		C1orf21
		GAP43
		DYRK2
		ITPR1
		BCL11A
		PAX3
		RSBN1
		DNAJB9
		BCL2L11
		NOX4
		INSIG1
		GATA6
		RGS3
		CPEB4
		HIVEP1
		DPP10
		SMAD7
		PPP1R12A
		HAND2
		PHF15
		SYNJ1
		SLC32A1
		PER2
		SMAD6
		TWIST1
		GOLGA3
		SESN3
		TRIO
		PIK3AP1
		PDE4D
		GLCE
		ARMC1
		CACNA1H
		EPC2
		SUV420H1
		C6orf62
	hsa-miR-32	MYLIP
		KIF5B

		RAB14
		NPC1
		HERC2
		ADAM23
		CNNM4
		PAIP1
		CTTNBP2
		FBXO33
		SDC2
		TAF15
		IRS2
		PHF3
		NELF
		NETO1
		PCDH10
		NOTCH1
		ZNF521
		PCDH9
		PRKAR2B
		TOP1
		SSBP3
		STAG2
		TBX3
		LRRC4
		CPEB1
		PLEKHM1
		ZDHHC21
		FSTL1
		LRRC20
		C18orf1
		ULK1
		NEGR1
		FBXO28
		CREB1
	hsa-miR-98	PBX3
		MAP4K3
		RDH10
		FGF11
		KIAA1274
		PRRX1
		ULK2
		SCD
		ABCB9
		SYT7
		SCN4B
		GHR
		PAPPA

		SLC16A14
		SEC24C
		COL4A2
Sub 3	hsa-miR-15b	EPB41L4B
		RECK
		PCDHA13
		PCDHA5
		PCDHA4
		PCDHA12
		PCDHA11
		PCDHA10
		PCDHAC2
		PCDHAC1
		PCDHA6
		PCDHA7
		PCDHA8
		PCDHA2
		PCDHA1
		PCDHA3
		PCDHA9
		ESRRA
		MAP4
		MAP2K1
		ADAMTSL3
		KCNJ2
		SPTLC1
		NLGN1
		KPNA4
		DEDD
		PDCD4
		FBXO21
		NFATC3
		ARL2
		CECR6
		CACNB1
		CLOCK
		CHORDC1
		ABCG4
		WDR47
		TGIF2
		ASPH
		SLC6A4
		ZNF423
		EIF4E
		KLC2
		WISP1

		TRIP10
		GRIN1
		MNT
Sub 4	hsa-let-7f	SMARCAD1
		BZW1
		EIF2C3
		NPHP3
		TRIM41
		CYP19A1
		ZBTB5
		TGFBR1
		CCR7
		RGAG1
		EFHD2
		ADIPOR2
		NPEPL1
	hsa-miR-335	SORCS1
		ETF1
		HAND1
		PCDH9
		CRIM1
		F13A1
		NR4A3
		ZMPSTE24
		ISL1
		CALU
		DAAM1
		CHFR
		RPRM
		HOXD8
		STIM2
		RASA1
		LPHN2
		SNIP1
		RNF141
		SRPR
		NRXN1
		ADCY3
		FMR1
		CCNT2
		EPHA4
		TPST1
		MAX
		UBE2G1
		ATP1B1
		KDELR1

		ADAM19
		SYN2
		CDH11
		MOSPD1
		PSD3
		BCL11B
		MBNL1
		EEF2K
		MAP3K2
		MAP2
		PRKD1
		SP1
		CRKL
		BPIL1
		PGF
		N4BP1
		MYO1D
		HPCAL4
		HIC2
**Sub 5**	hsa-miR-361-3p	None
	hsa-miR-362-5p	None
